# Supplementary material for: Investigating a relation between amyloid beta plaque burden and accumulated neurotoxicity caused by amyloid beta oligomers
Source: Med Biol Eng Comput. 2026 Jun 30;64(7):2747–68. doi: 10.1007/s11517-026-03606-z (PMC13468985; doi:10.1007/s11517-026-03606-z)
Supplement: Supplementary file 1 [file 11517_2026_3606_MOESM1_ESM.pdf]

Investigating a Relation between Amyloid Beta Plaque Burden and Accumulated Neurotoxicity Caused by Amyloid Beta Oligomers

Andrey V Kuznetsov

Department of Mechanical and Aerospace Engineering, North Carolina State University,

Raleigh, NC 27695-7910, USA; e-mail: avkuznet@ncsu.edu

Supplemental Materials

S1. Numerical Solution

The system of differential equations, Eqs. (1)-(4), subject to the initial conditions of Eq. (5), was integrated numerically using the ODE45 solver in MATLAB (R2024a, MathWorks, Natick, MA, USA). This solver employs an adaptive step-size Runge–Kutta scheme and was selected for its reliability and accuracy. To guarantee high numerical precision, both the relative and absolute tolerances (RelTol and AbsTol) were set to 10^{-10}.

S2. Supplementary figures

(a)

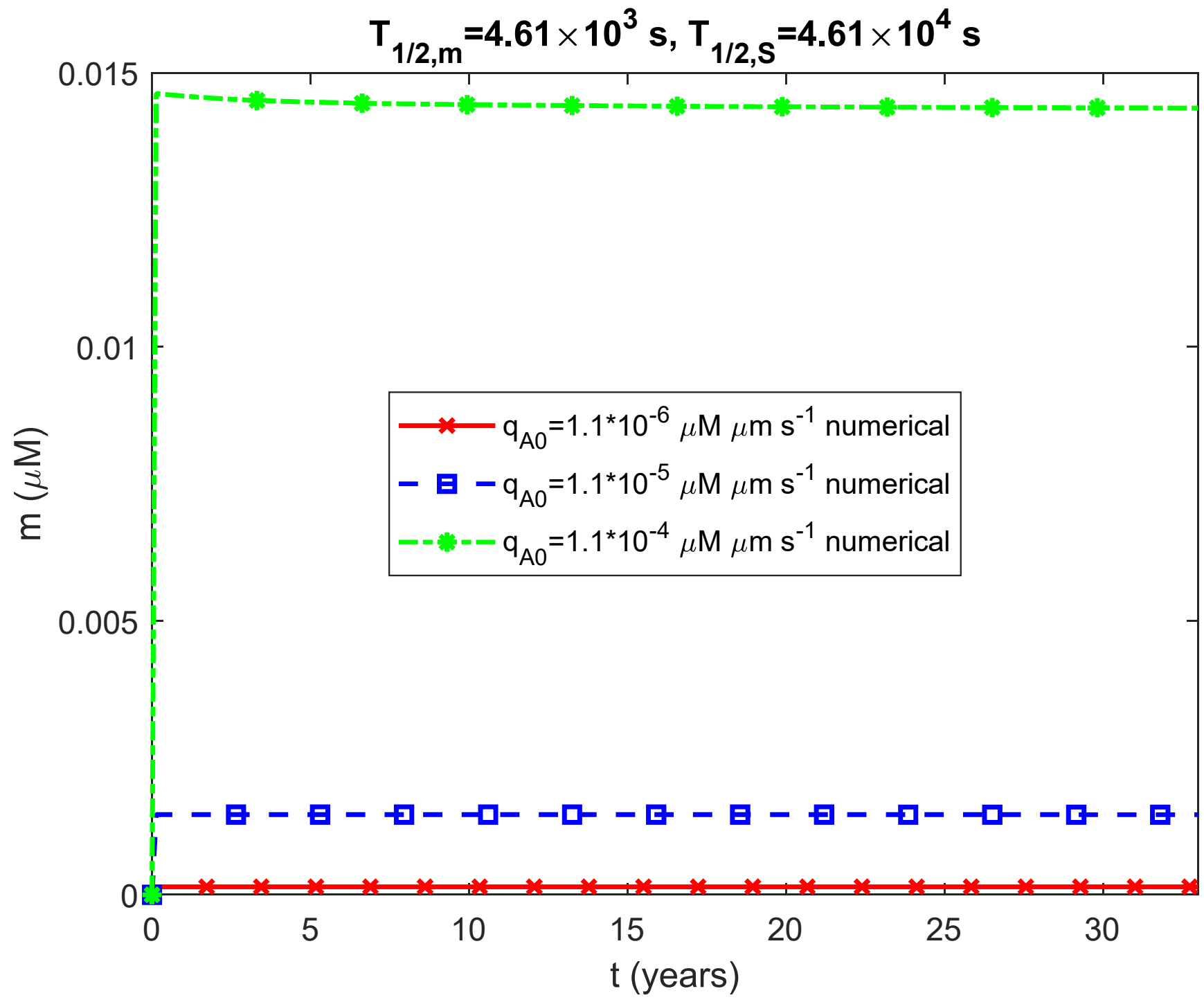
T1/2,m=4.61×10^3 s, T1/2,S=4.61×10^4 s
0.015
0.01
0.005
0
m (μM)
qA0=1.1*10^-6 μM μm s^-1 numerical
qA0=1.1*10^-5 μM μm s^-1 numerical
qA0=1.1*10^-4 μM μm s^-1 numerical
0 5 10 15 20 25 30
t (years)

(b)

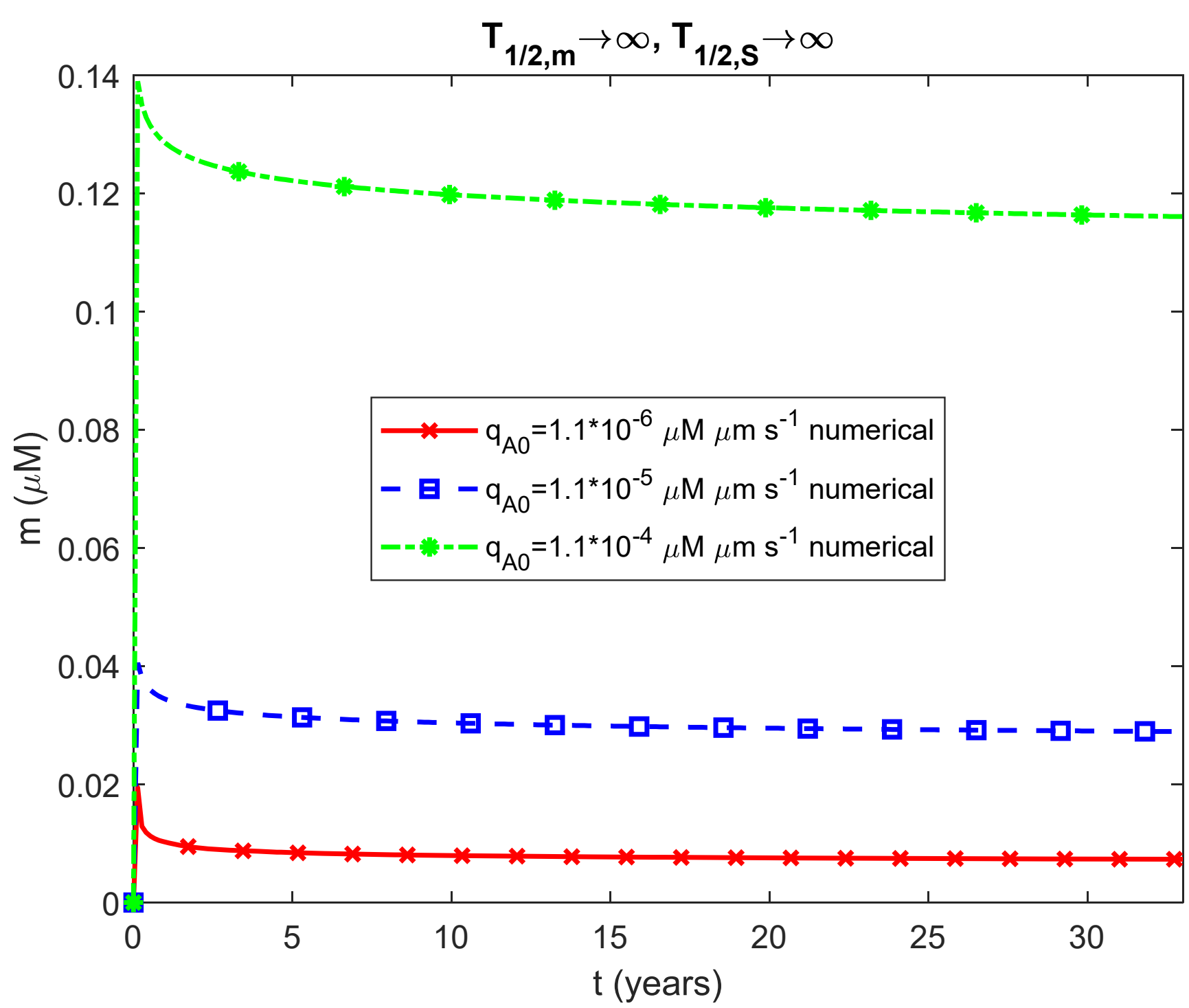
T1/2,m→∞, T1/2,S→∞
0.14
0.12
0.1
0.08
0.06
0.04
0.02
0
m (μM)
qA0=1.1*10^-6 μM μm s^-1 numerical
qA0=1.1*10^-5 μM μm s^-1 numerical
qA0=1.1*10^-4 μM μm s^-1 numerical
0 5 10 15 20 25 30
t (years)

Fig. S1. Molar concentration of free Aβ monomers as a function of time, $m(t)$. The case in which oligomer dissociation into monomers is accounted for ($k_{d1} = 3\times10^{-6}$ s^{-1}, $k_{d2} = 1.94\times10^{-5}$ μM^{-1} s^{-1}) and fibril fragmentation is neglected ($k_{-} = 0$).

(a)

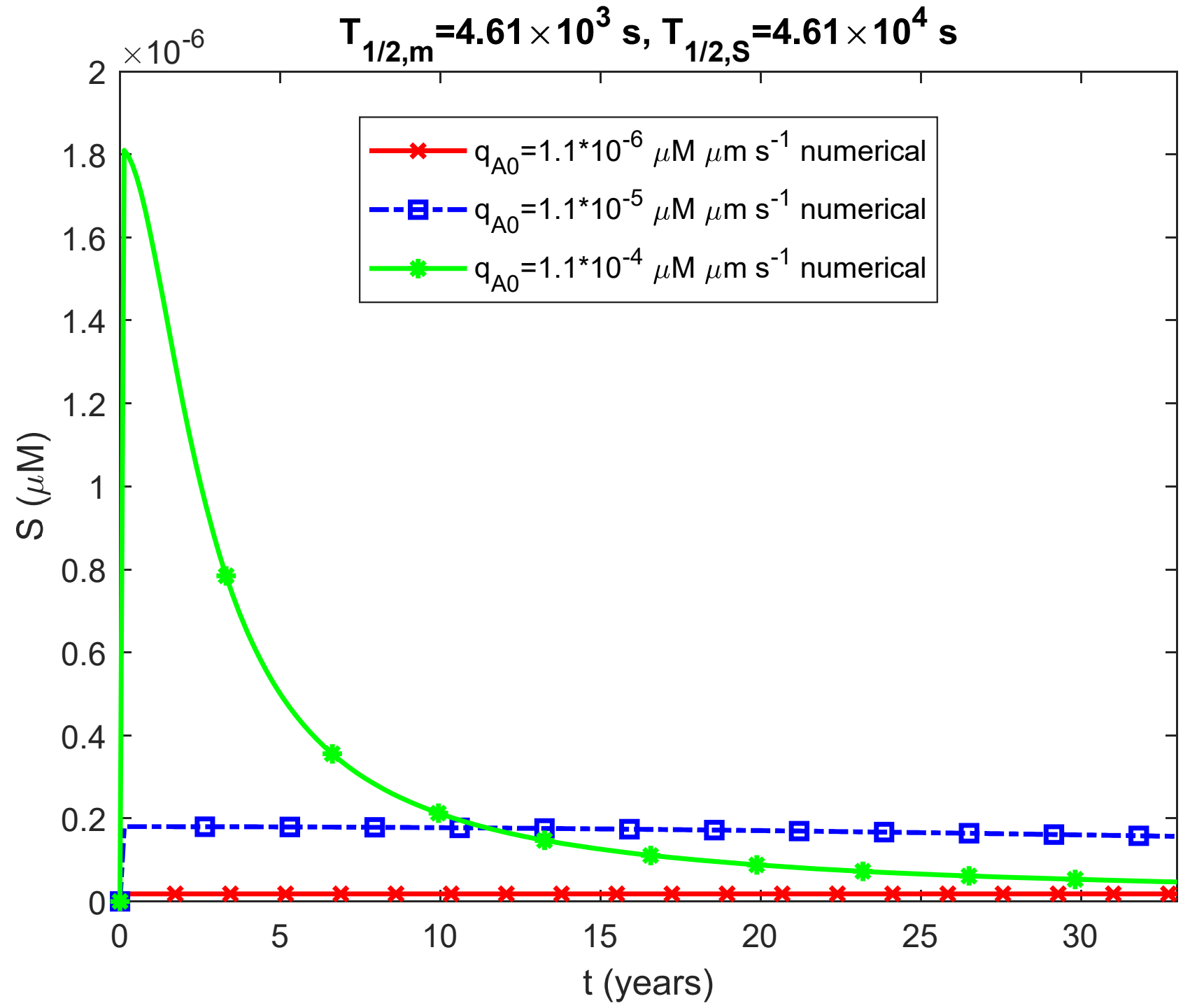

(b)

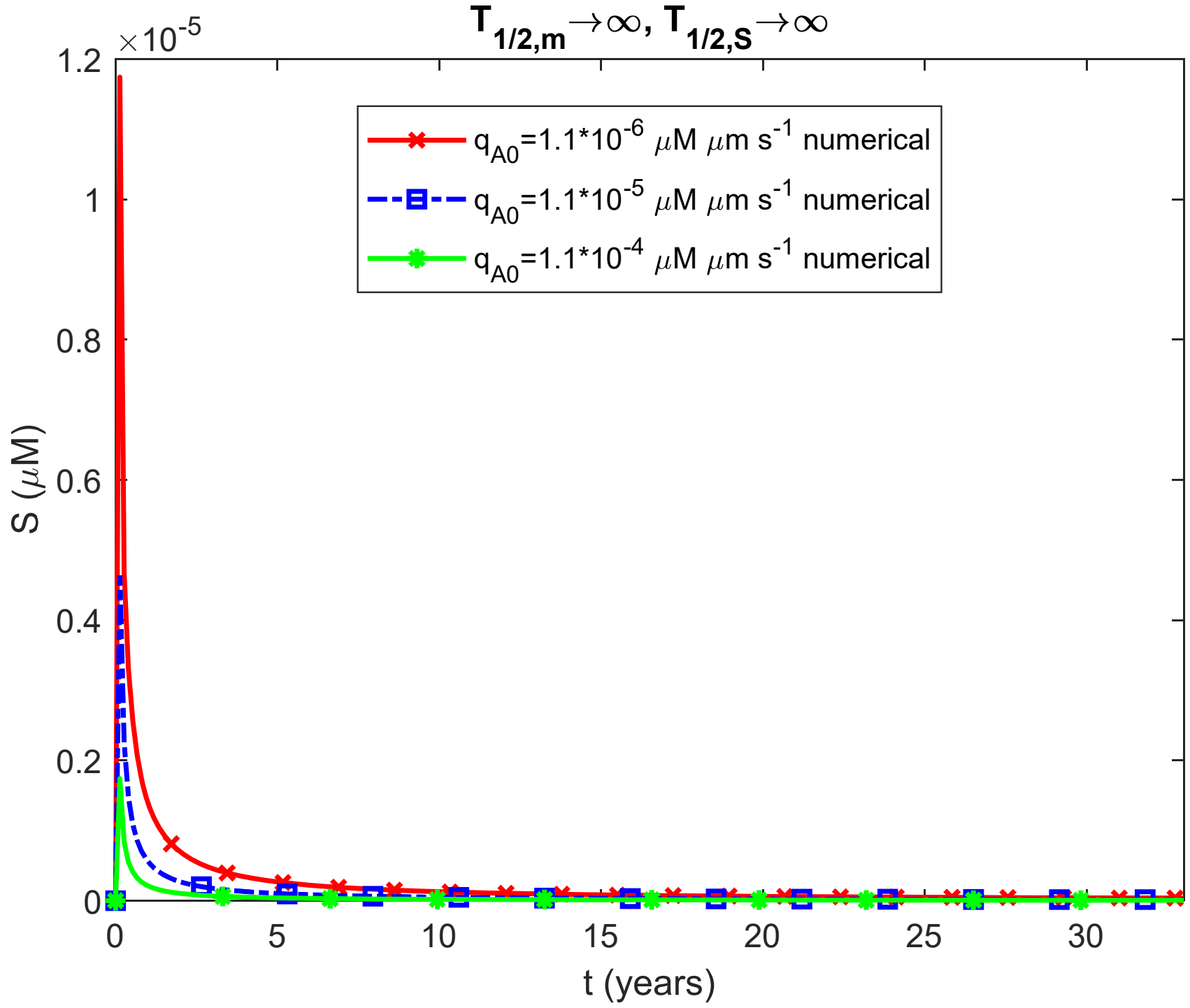

Fig. S2. Molar concentration of free on-pathway Aβ oligomers as a function of time, $S(t)$. The case in which oligomer dissociation into monomers is accounted for ($k_{d1} = 3\times10^{-6}$ s^{-1}, $k_{d2} = 1.94\times10^{-5}$ μM^{-1} s^{-1}) and fibril fragmentation is neglected ($k_{-} = 0$).

(a)

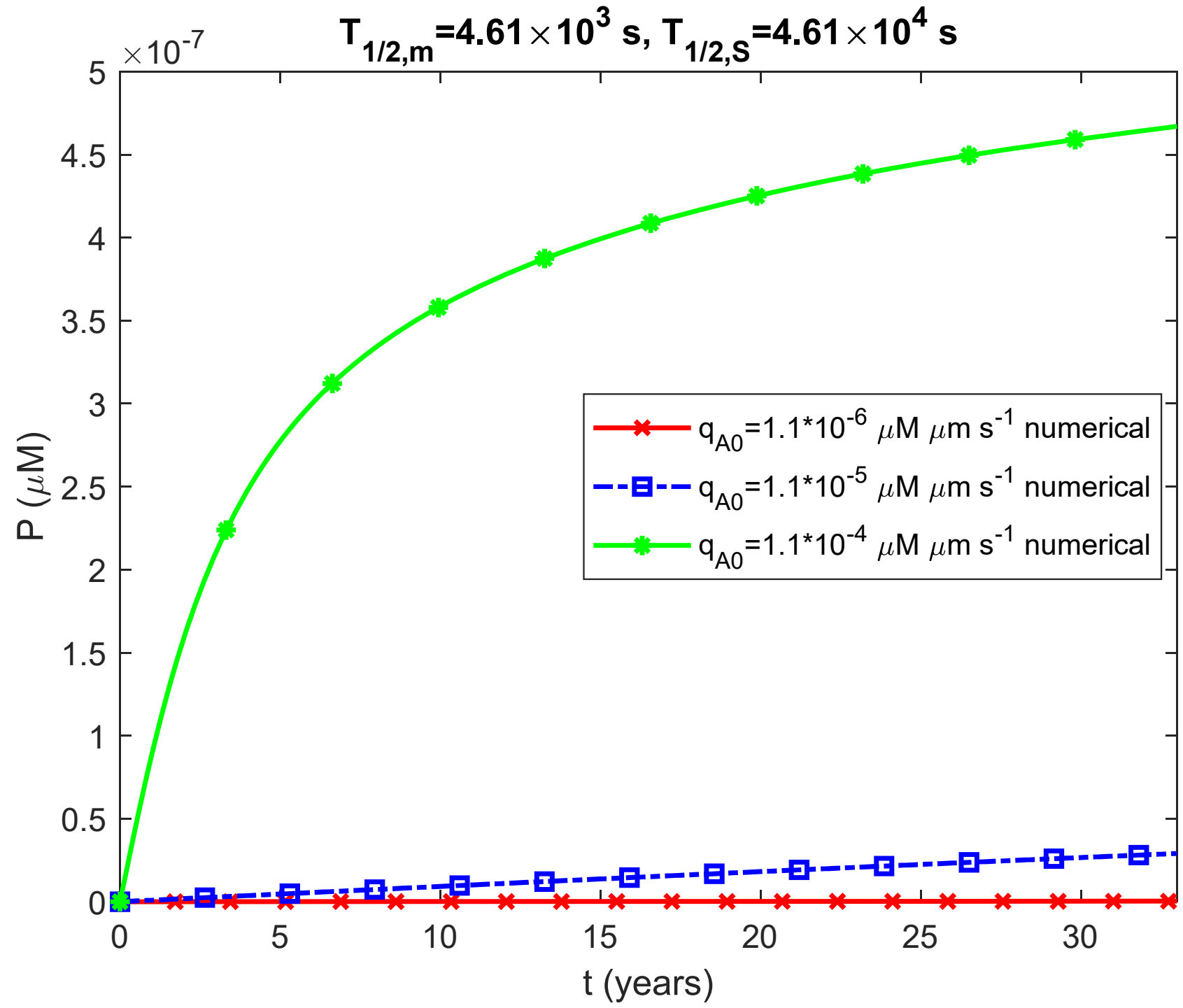

T1/2,m=4.61×10^3 s, T1/2,S=4.61×10^4 s
×10^-7
P (μM)
t (years)
qA0=1.1*10^-6 μM μm s^-1 numerical
qA0=1.1*10^-5 μM μm s^-1 numerical
qA0=1.1*10^-4 μM μm s^-1 numerical

(b)

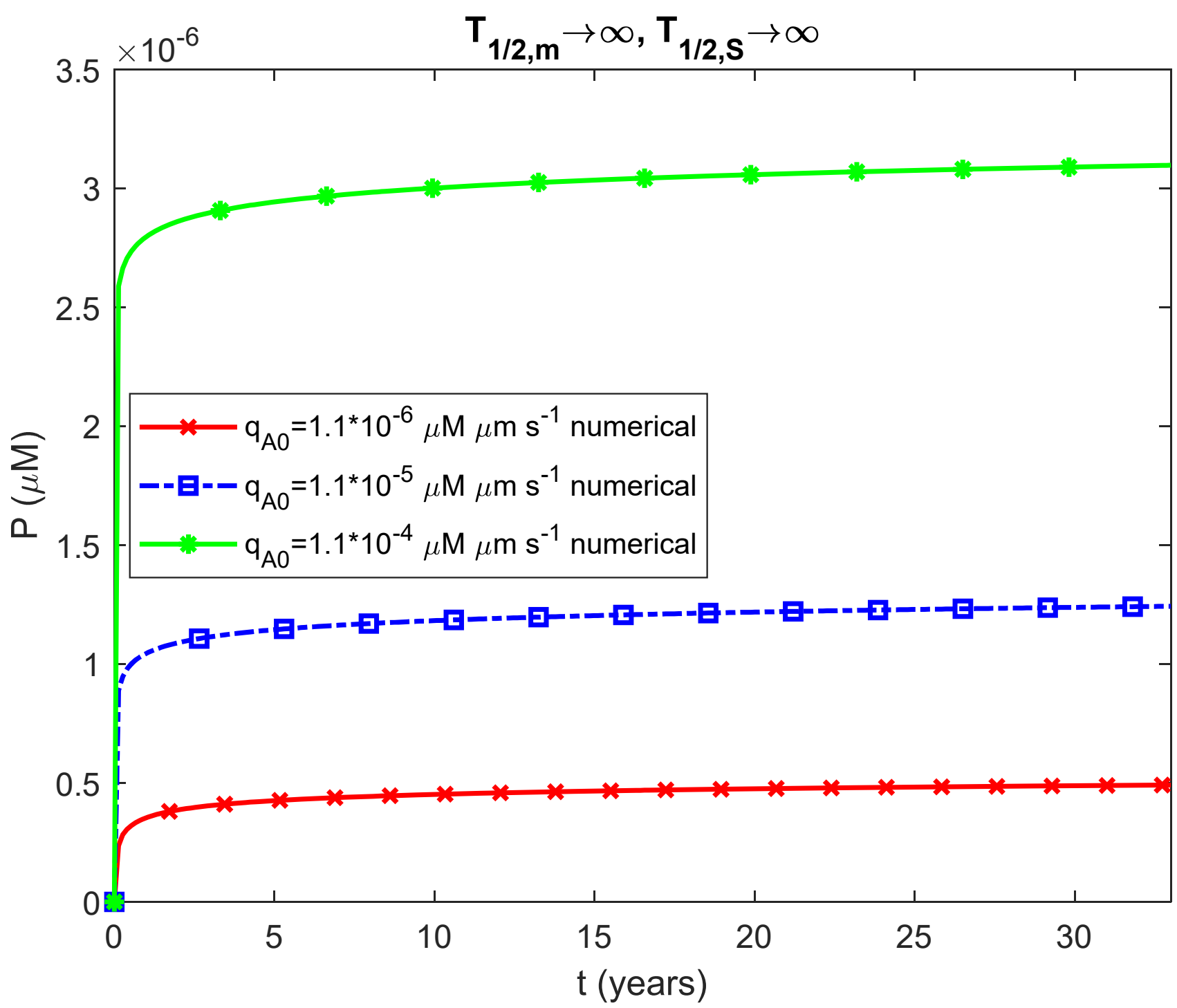

T1/2,m→∞, T1/2,S→∞
×10^-6
P (μM)
t (years)
qA0=1.1*10^-6 μM μm s^-1 numerical
qA0=1.1*10^-5 μM μm s^-1 numerical
qA0=1.1*10^-4 μM μm s^-1 numerical

Fig. S3. Molar concentration of Aβ fibrillar species of varying length as a function of time, $P(t)$. The case in which oligomer dissociation into monomers is accounted for ($k_{d1} = 3\times10^{-6}$ s^{-1}, $k_{d2} = 1.94\times10^{-5}$ μM^{-1} s^{-1}) and fibril fragmentation is neglected ($k_- = 0$).

(a)

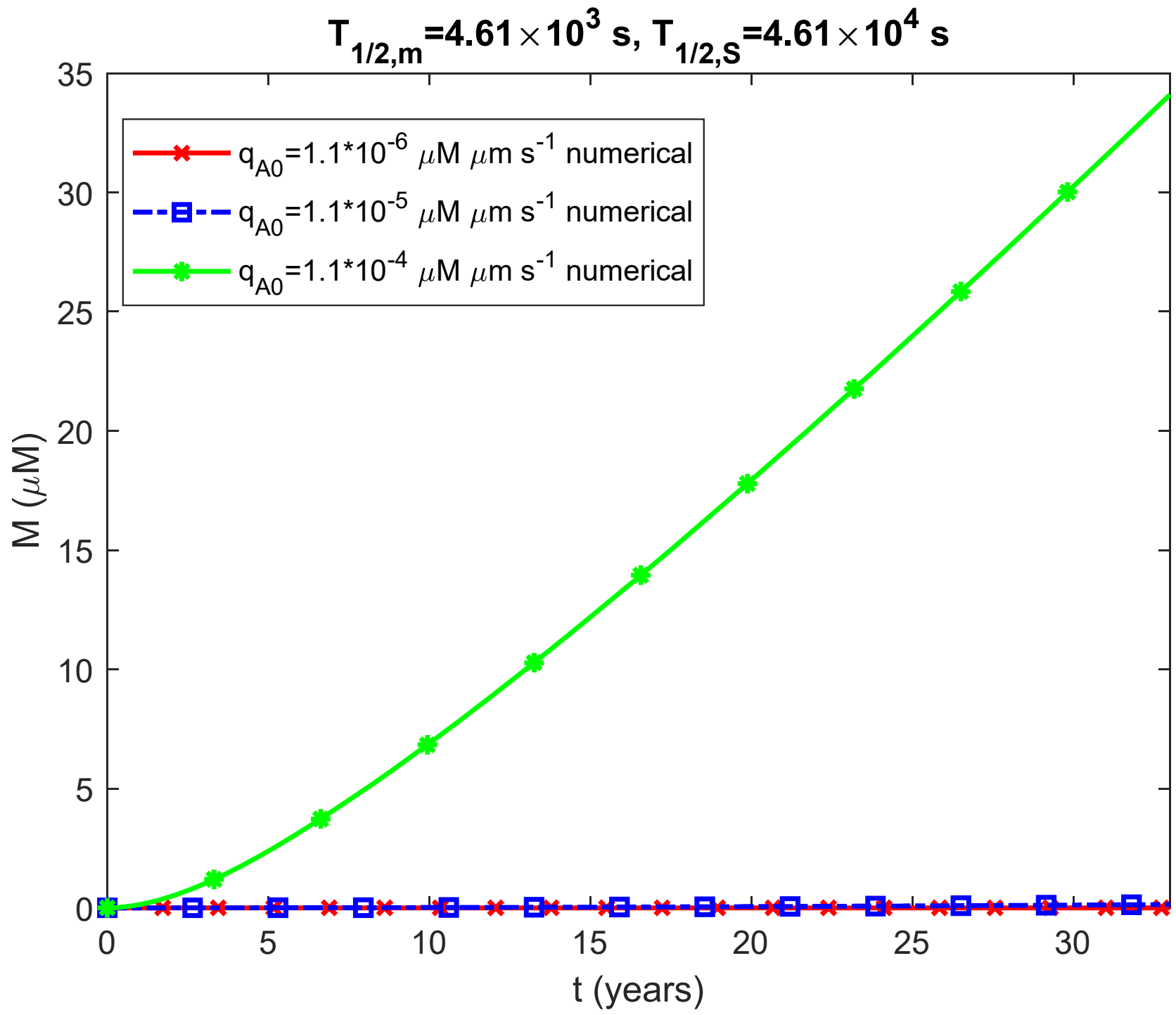

(b)

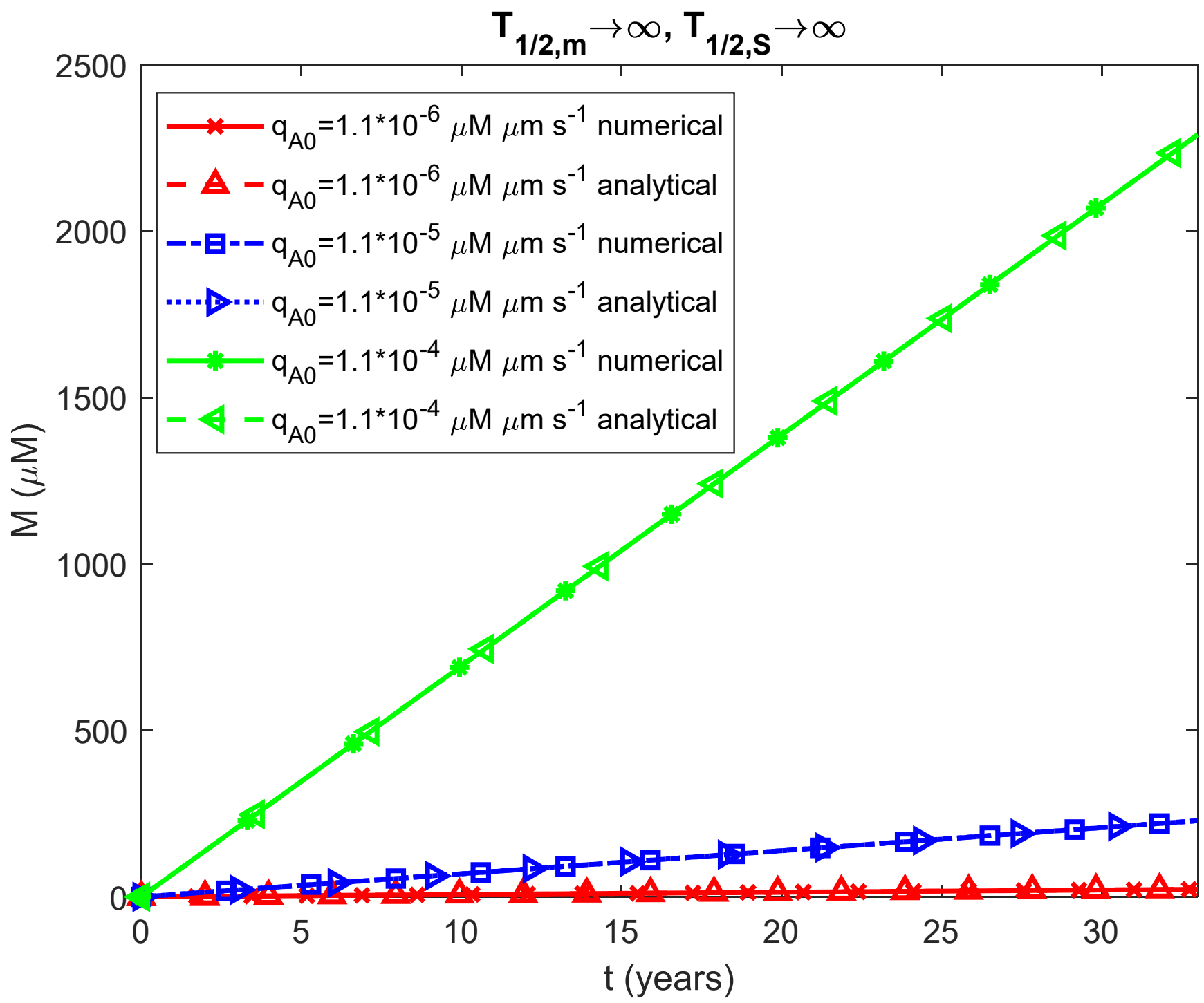

Fig. S4. Molar concentration of Aβ monomers incorporated into fibrillar species of varying length as a function of time, $M(t)$. The case in which oligomer dissociation into monomers is accounted for ($k_{d1} = 3\times10^{-6}$ s^{-1}, $k_{d2} = 1.94\times10^{-5}$ μM^{-1} s^{-1}) and fibril fragmentation is neglected ($k_{-} = 0$).

(a)

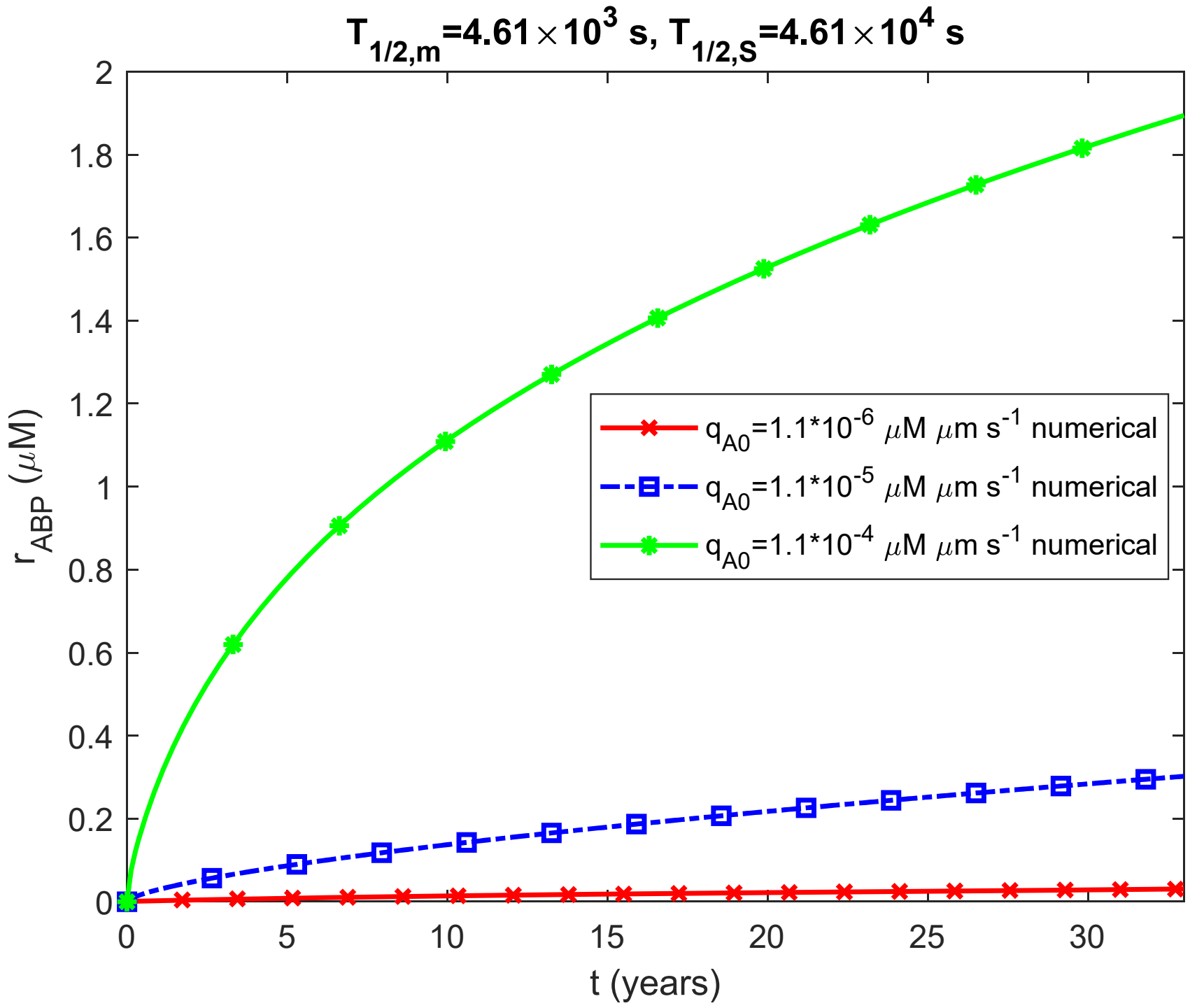

$T_{1/2,m}$=4.61×10^3 s, $T_{1/2,S}$=4.61×10^4 s
q_{A0}=1.1*10^{-6} μM μm s^{-1} numerical
q_{A0}=1.1*10^{-5} μM μm s^{-1} numerical
q_{A0}=1.1*10^{-4} μM μm s^{-1} numerical
r_{ABP} (μM)
t (years)

(b)

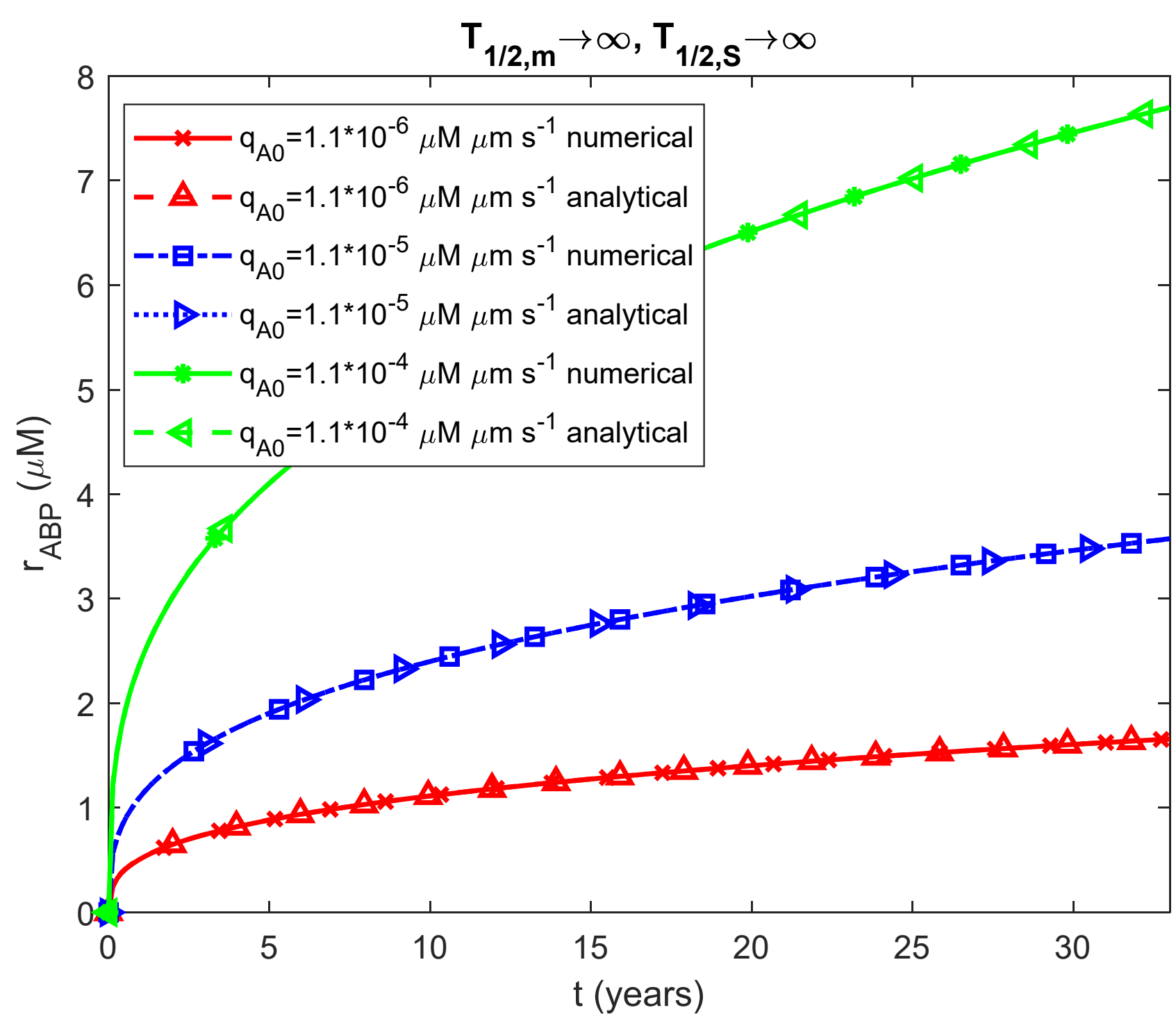

$T_{1/2,m}$→∞, $T_{1/2,S}$→∞
q_{A0}=1.1*10^{-6} μM μm s^{-1} numerical
q_{A0}=1.1*10^{-6} μM μm s^{-1} analytical
q_{A0}=1.1*10^{-5} μM μm s^{-1} numerical
q_{A0}=1.1*10^{-5} μM μm s^{-1} analytical
q_{A0}=1.1*10^{-4} μM μm s^{-1} numerical
q_{A0}=1.1*10^{-4} μM μm s^{-1} analytical
r_{ABP} (μM)
t (years)

Fig. S5. Radius of the Aβ plaque as a function of time, $r_{ABP}(t)$. The case in which oligomer dissociation into monomers is accounted for ($k_{d1}=3\times10^{-6}$ s^{-1}, $k_{d2}=1.94\times10^{-5}$ μM^{-1} s^{-1}) and fibril fragmentation is neglected ($k_{-}=0$).

(a)

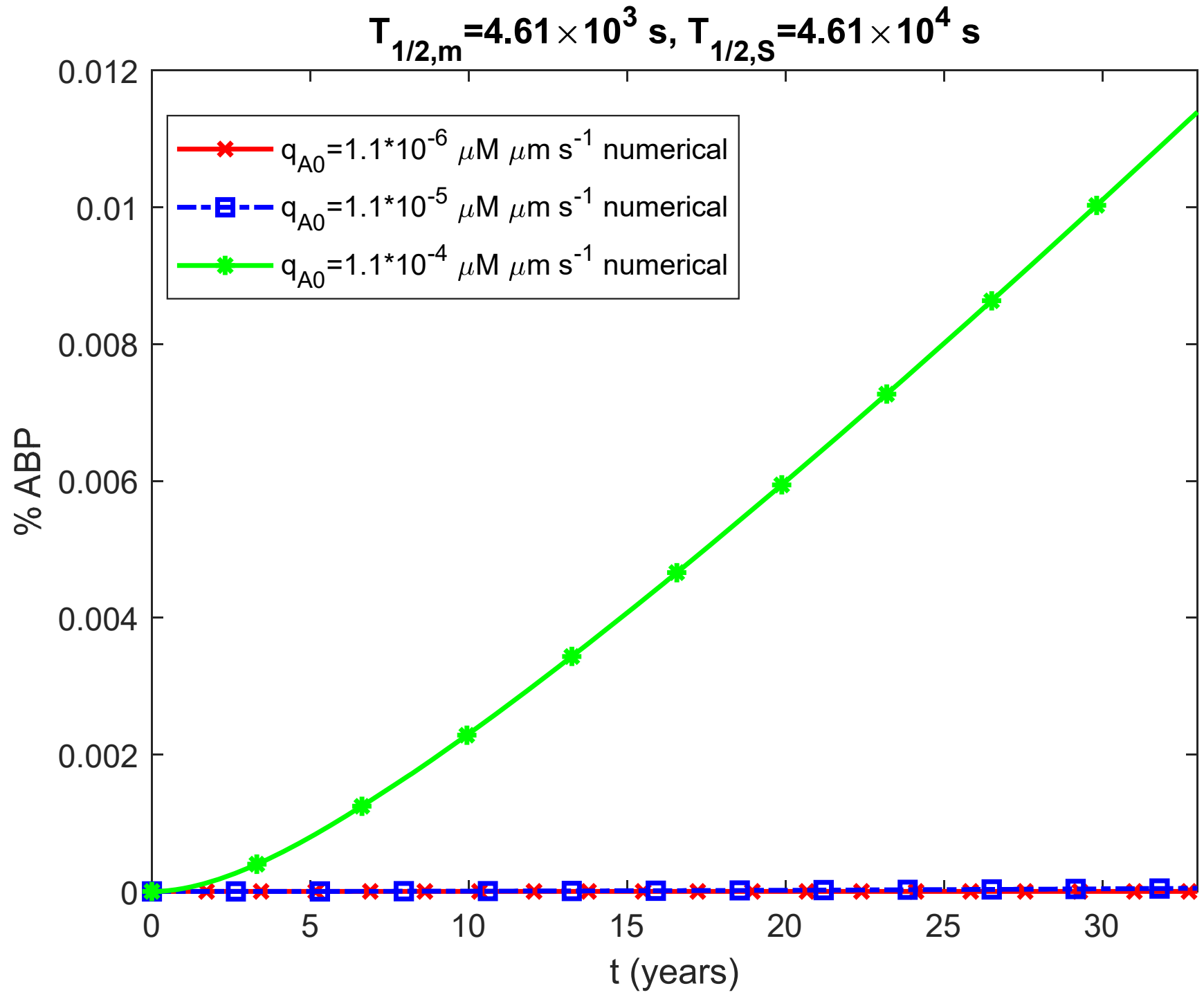

(b)

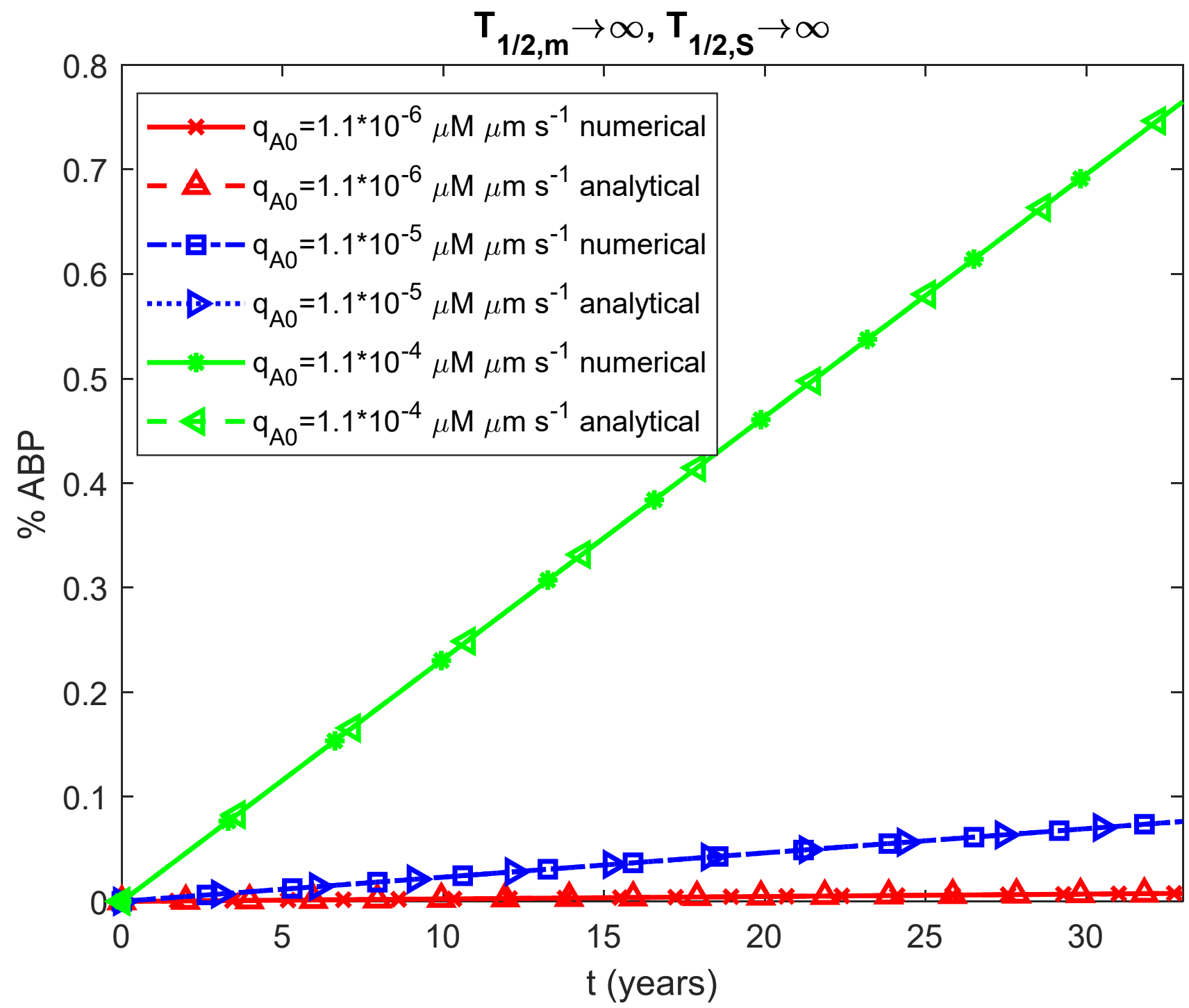

Fig. S6. Percentage of the CV occupied by the Aβ plaque as a function of time. The case in which oligomer dissociation into monomers is accounted for ($k_{d1} = 3\times10^{-6}$ s^{-1}, $k_{d2} = 1.94\times10^{-5}$ μM^{-1} s^{-1}) and fibril fragmentation is neglected ($k_{-} = 0$).

(a)

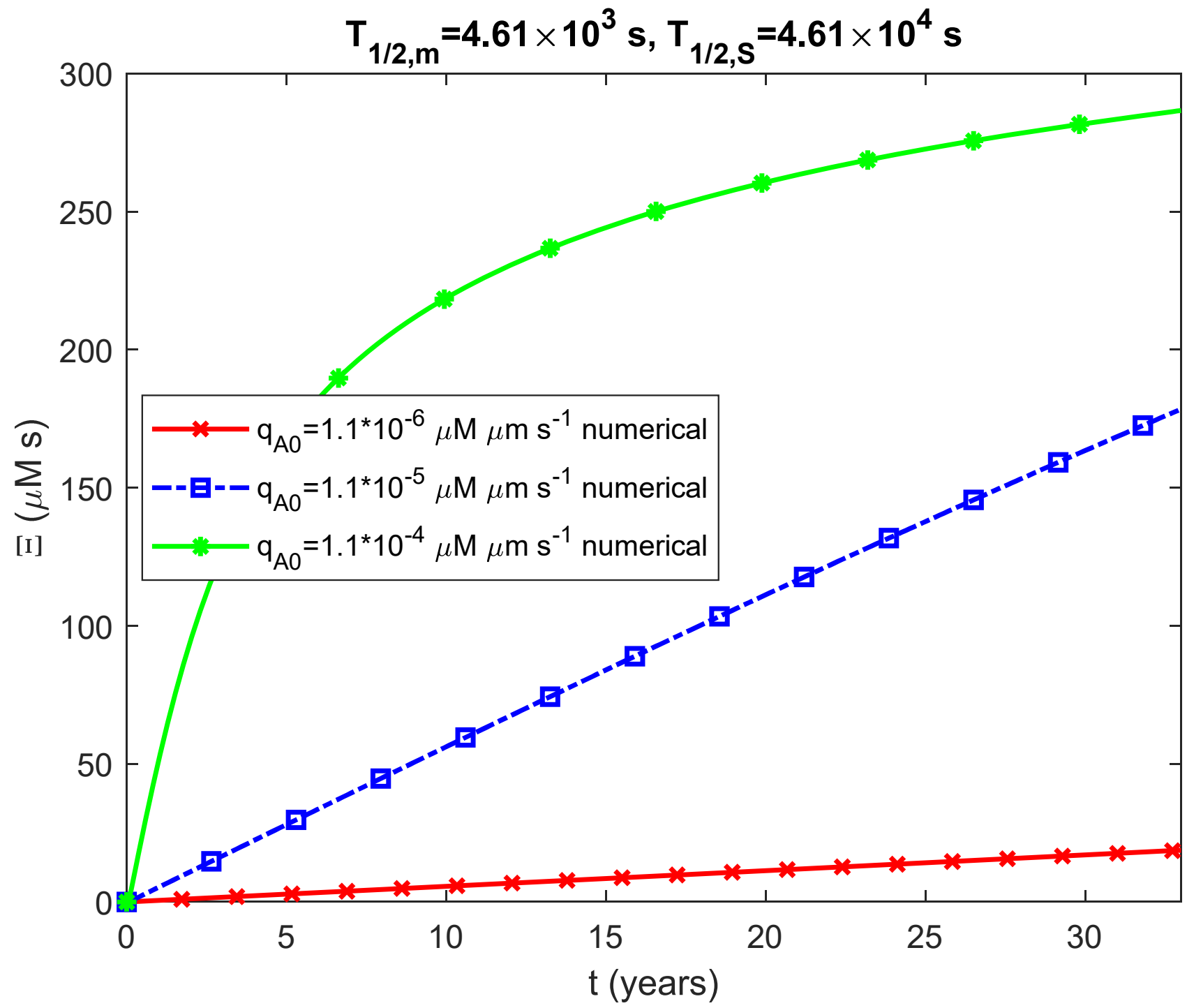

$T_{1/2,m}$=4.61×10^3 s, $T_{1/2,S}$=4.61×10^4 s
Ξ (μM s)
t (years)
q_{A0}=1.1*10^{-6} μM μm s^{-1} numerical
q_{A0}=1.1*10^{-5} μM μm s^{-1} numerical
q_{A0}=1.1*10^{-4} μM μm s^{-1} numerical

(b)

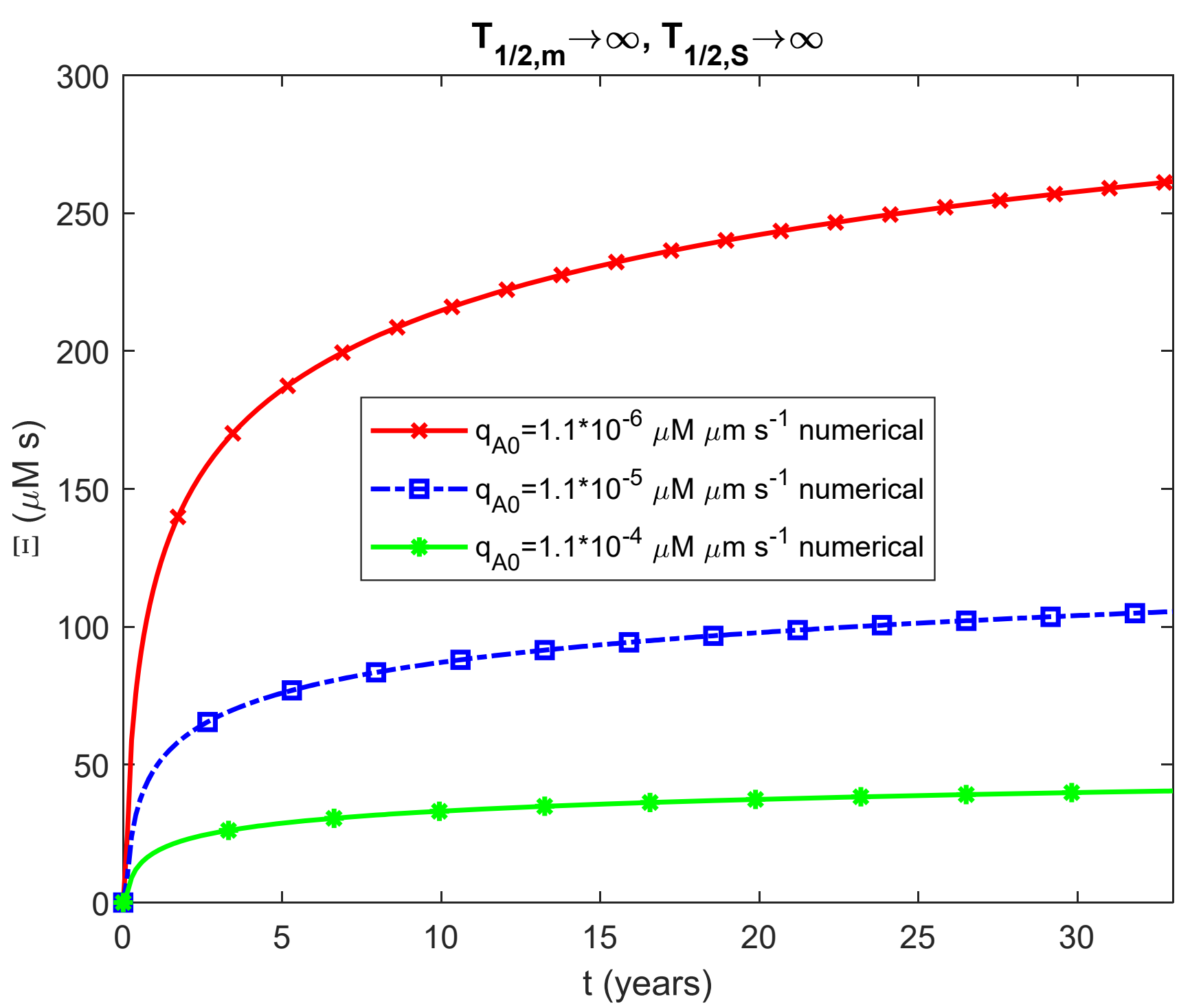

$T_{1/2,m}$→∞, $T_{1/2,S}$→∞
Ξ (μM s)
t (years)
q_{A0}=1.1*10^{-6} μM μm s^{-1} numerical
q_{A0}=1.1*10^{-5} μM μm s^{-1} numerical
q_{A0}=1.1*10^{-4} μM μm s^{-1} numerical

Fig. S7. Accumulated neurotoxicity as a function of time, $\Xi(t)$. The case in which oligomer dissociation into monomers is accounted for ($k_{d1} = 3\times10^{-6}$ s^{-1}, $k_{d2} = 1.94\times10^{-5}$ μM^{-1} s^{-1}) and fibril fragmentation is neglected ($k_{-} = 0$).

(a)

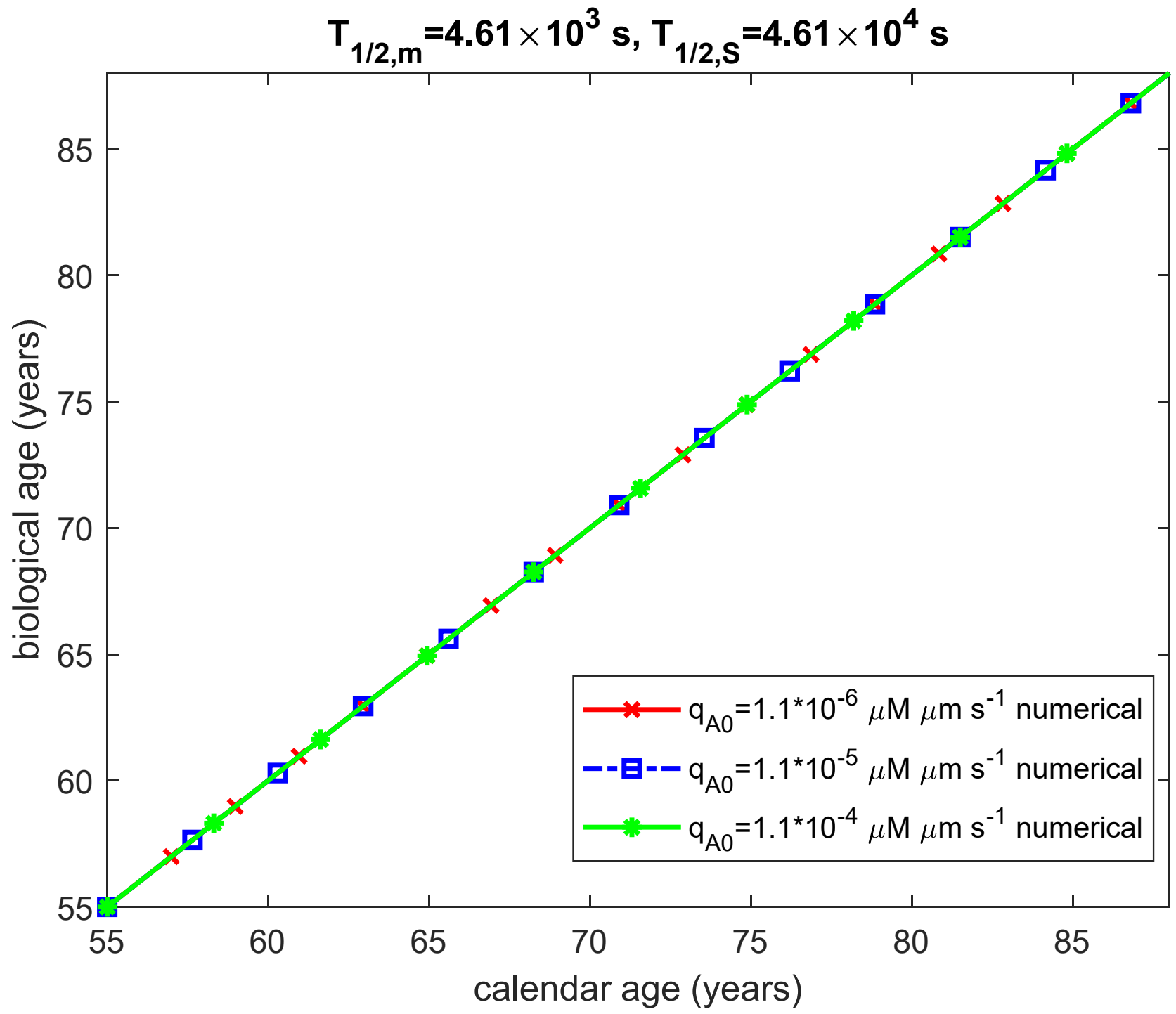

(b)

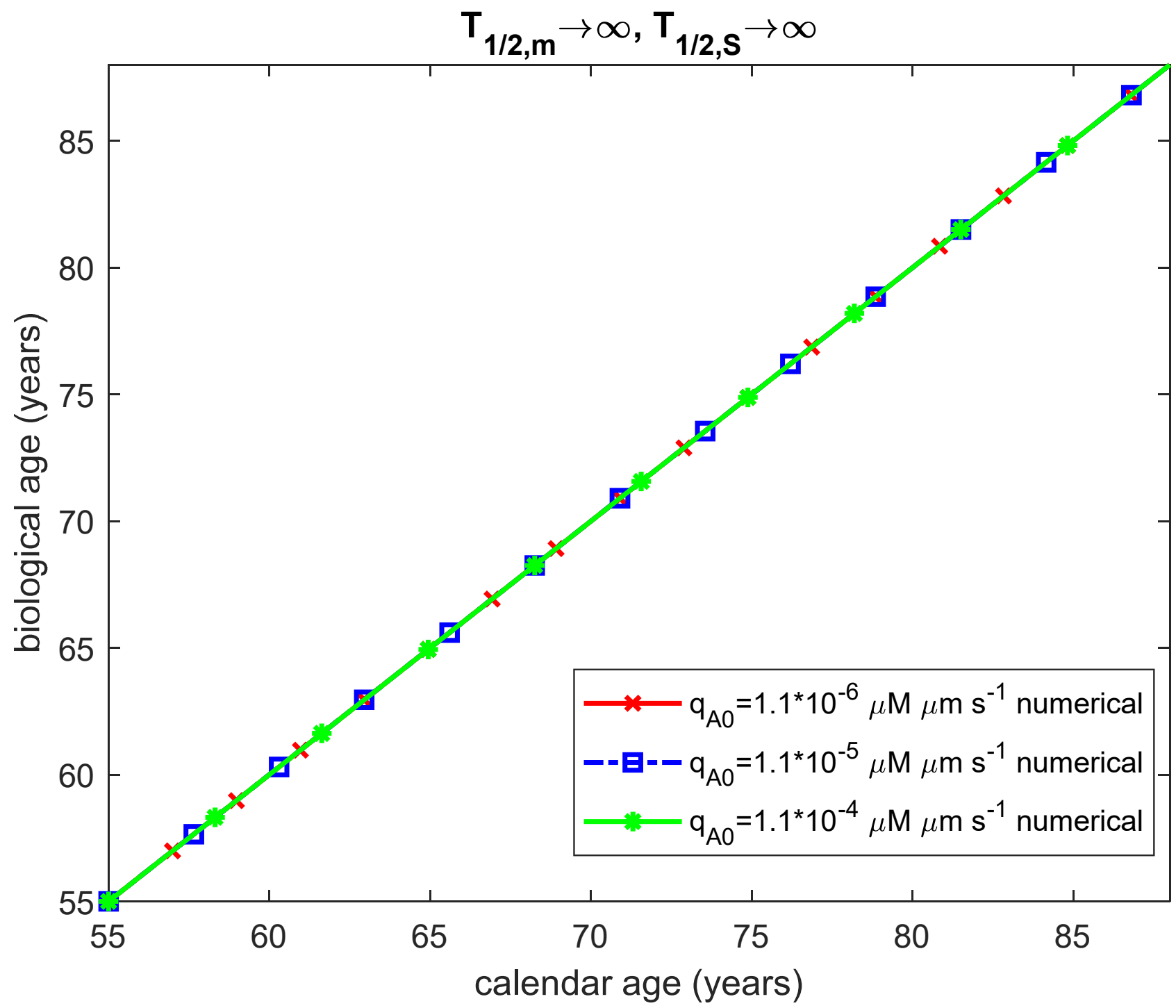

Fig. S8. Biological age as a function of calendar age. The case in which oligomer dissociation into monomers is accounted for ($k_{d1} = 3\times10^{-6}$ s^{-1}, $k_{d2} = 1.94\times10^{-5}$ μM^{-1} s^{-1}) and fibril fragmentation is neglected ($k_{-} = 0$).

(a)

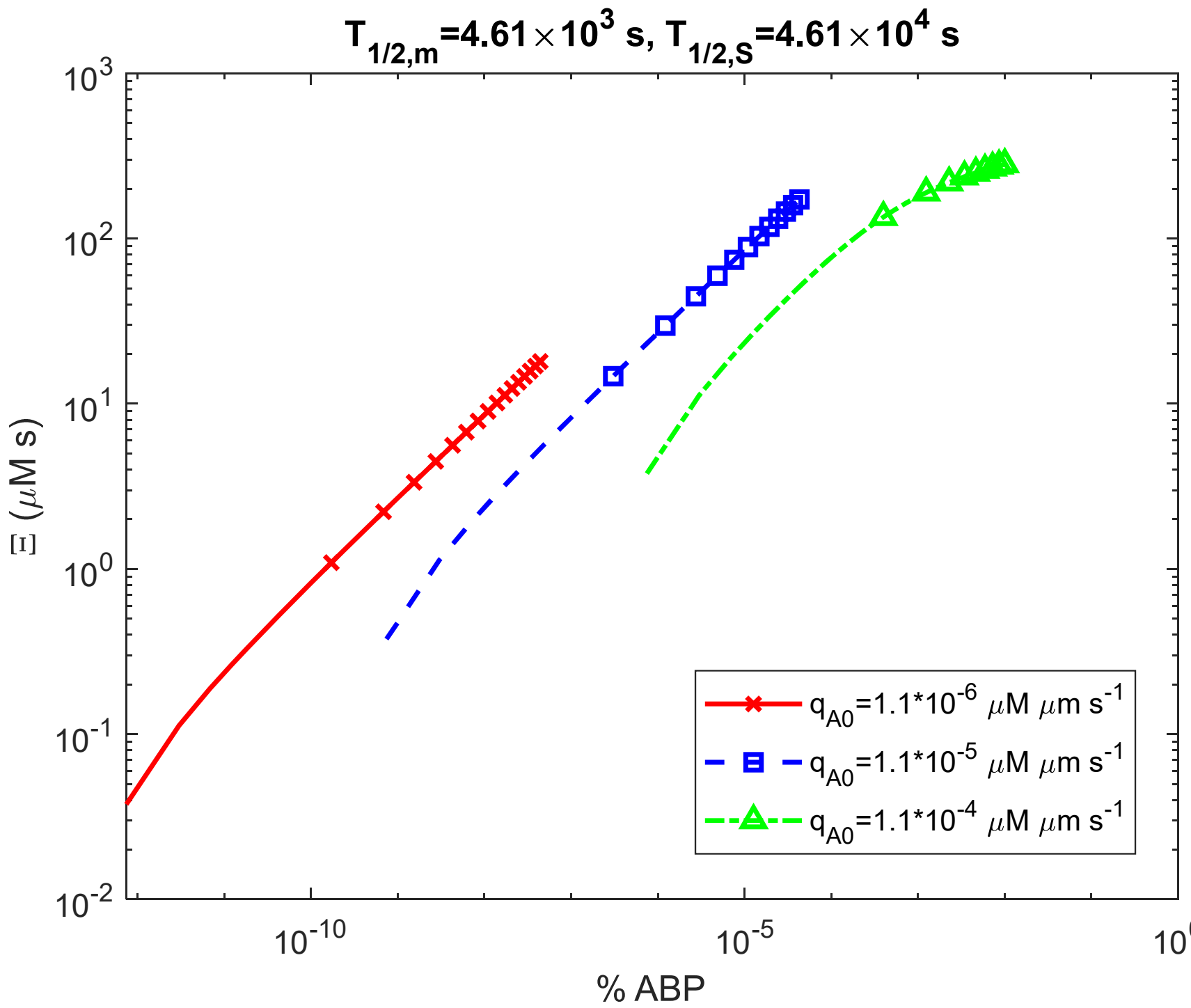

T1/2,m=4.61×10^3 s, T1/2,S=4.61×10^4 s
Ξ (μM s)
% ABP
qA0=1.1*10^-6 μM μm s^-1
qA0=1.1*10^-5 μM μm s^-1
qA0=1.1*10^-4 μM μm s^-1

(b)

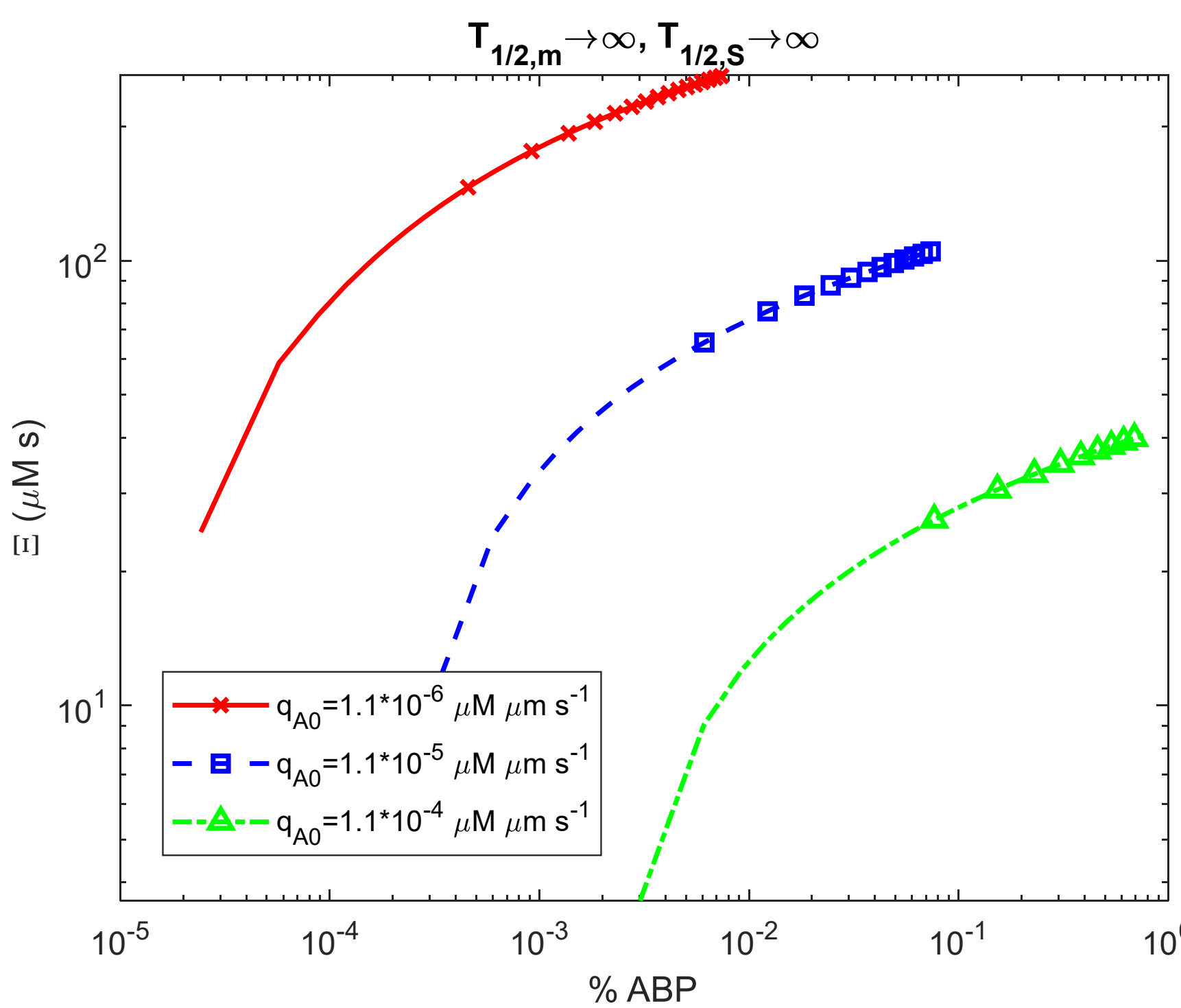

T1/2,m→∞, T1/2,S→∞
Ξ (μM s)
% ABP
qA0=1.1*10^-6 μM μm s^-1
qA0=1.1*10^-5 μM μm s^-1
qA0=1.1*10^-4 μM μm s^-1

Fig. S9. Accumulated neurotoxicity as a function of the percentage of the CV occupied by the Aβ plaque. The case in which oligomer dissociation into monomers is accounted for ($k_{d1}=3\times10^{-6}$ s^{-1}, $k_{d2}=1.94\times10^{-5}$ μM^{-1} s^{-1}) and fibril fragmentation is neglected ($k_{-}=0$).

(a)

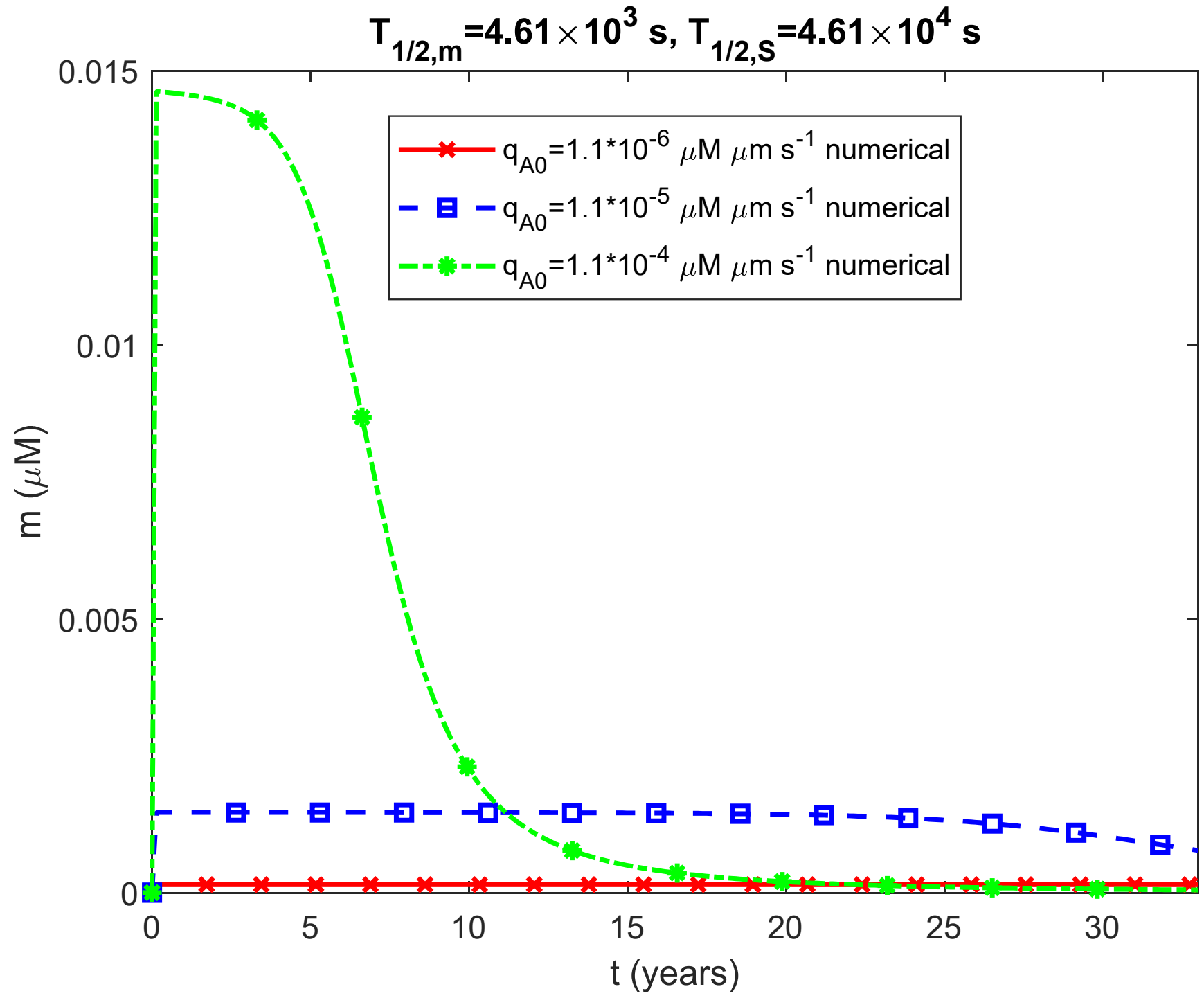

(b)

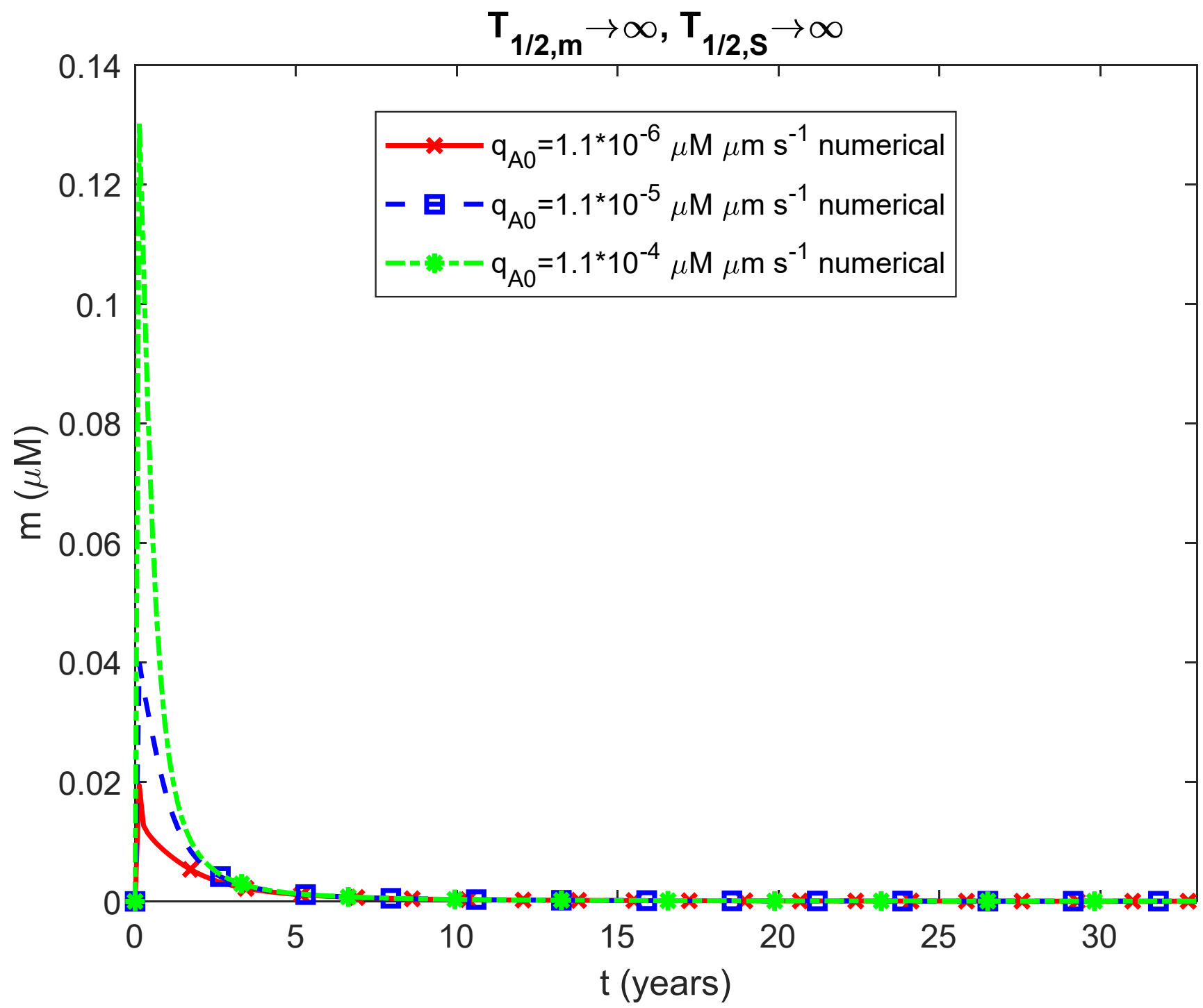

Fig. S10. Molar concentration of free Aβ monomers as a function of time, $m(t)$. The case in which both oligomer dissociation into monomers ($k_{d1} = 3\times10^{-6}$ s^{-1}, $k_{d2} = 1.94\times10^{-5}$ μM^{-1} s^{-1}) and fibril fragmentation ($k_{-} = 10^{-14}$ s^{-1}) are accounted for.

(a)

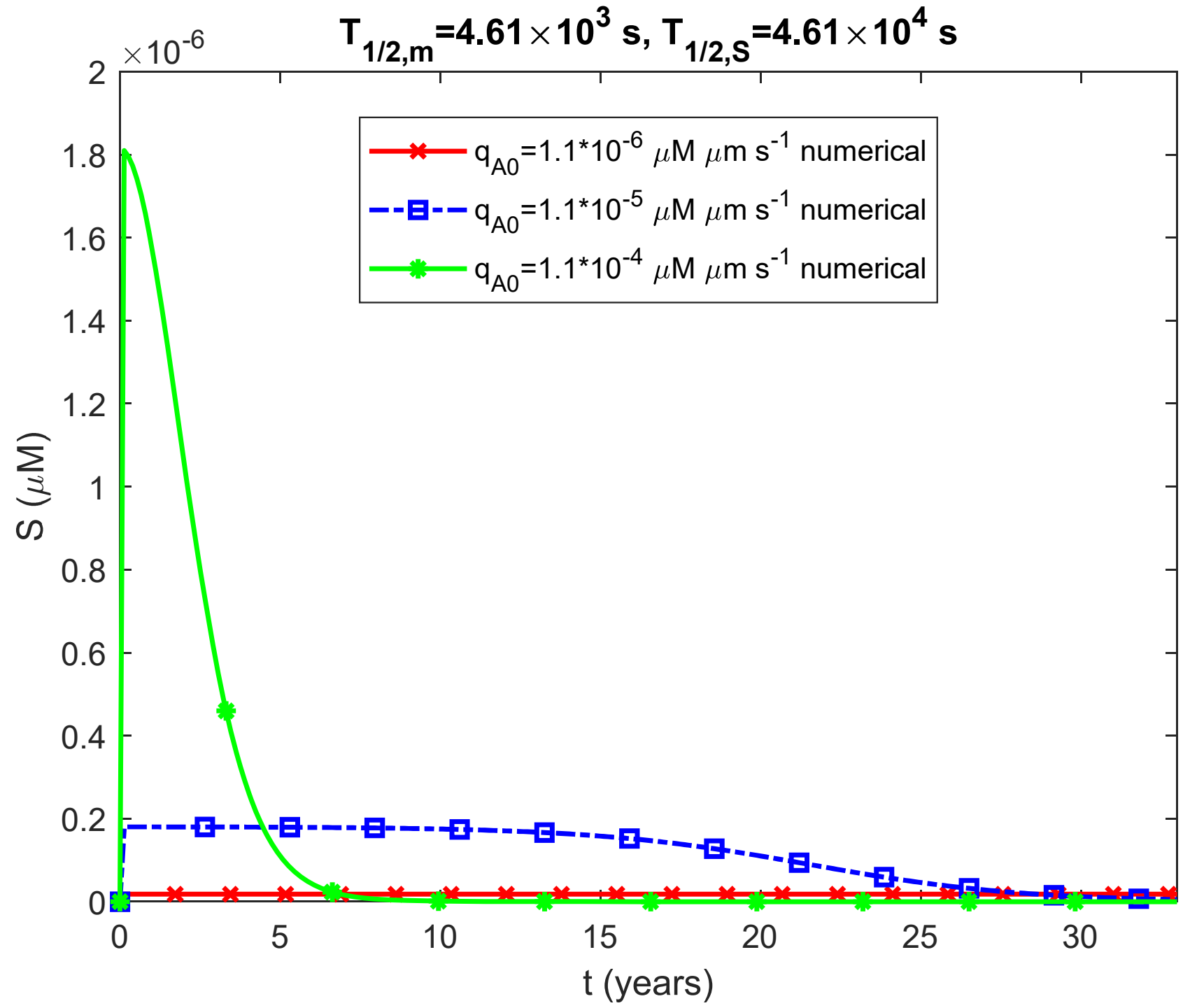
$T_{1/2,m}$=4.61×10^3 s, $T_{1/2,S}$=4.61×10^4 s
×10^-6
q_{A0}=1.1*10^-6 μM μm s^-1 numerical
q_{A0}=1.1*10^-5 μM μm s^-1 numerical
q_{A0}=1.1*10^-4 μM μm s^-1 numerical
S (μM)
t (years)

(b)

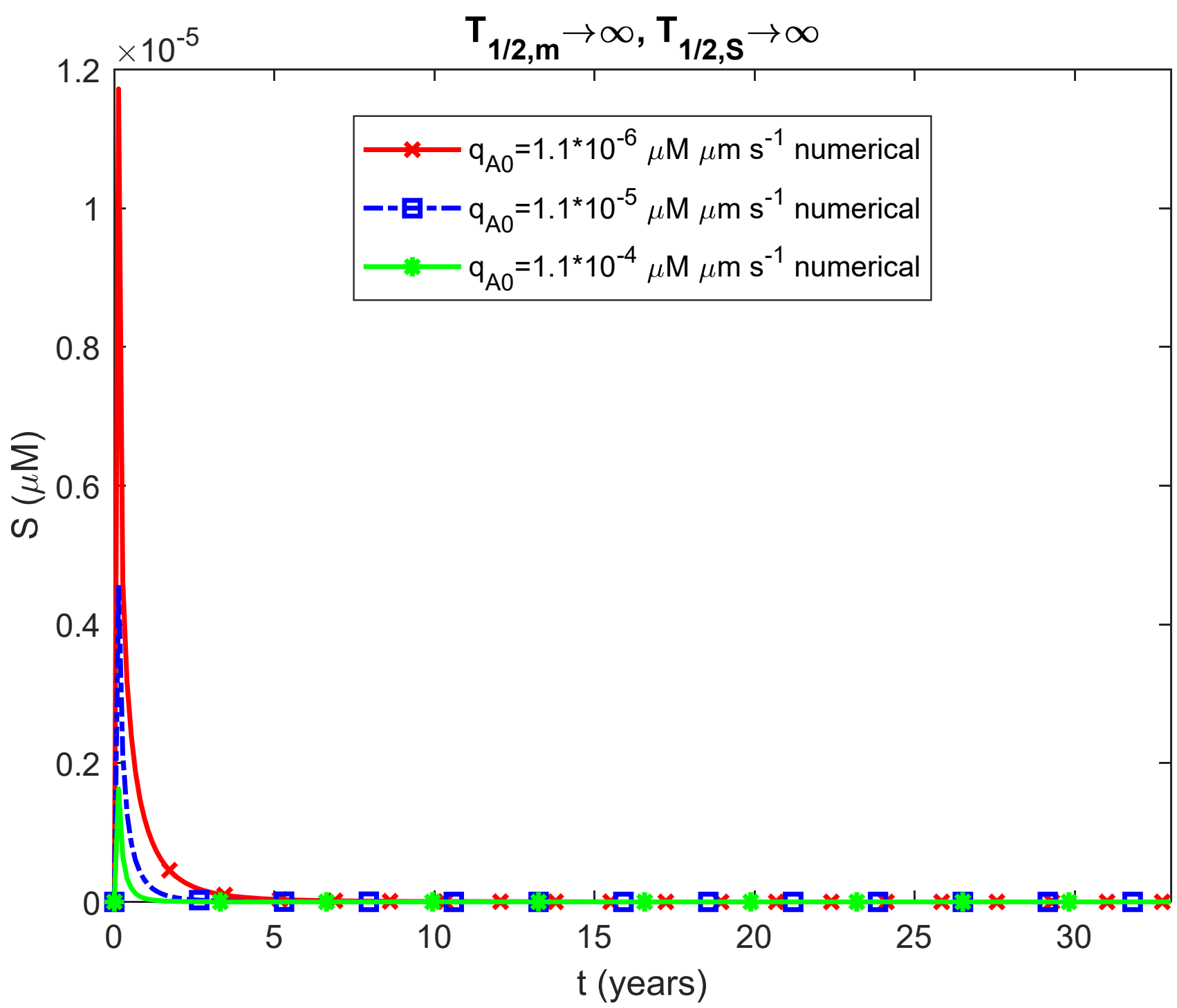
$T_{1/2,m}$→∞, $T_{1/2,S}$→∞
×10^-5
q_{A0}=1.1*10^-6 μM μm s^-1 numerical
q_{A0}=1.1*10^-5 μM μm s^-1 numerical
q_{A0}=1.1*10^-4 μM μm s^-1 numerical
S (μM)
t (years)

Fig. S11. Molar concentration of free on-pathway Aβ oligomers as a function of time, $S(t)$. The case in which both oligomer dissociation into monomers ($k_{d1}=3\times10^{-6}$ s^{-1}, $k_{d2}=1.94\times10^{-5}$ μM^{-1} s^{-1}) and fibril fragmentation ($k_{-}=10^{-14}$ s^{-1}) are accounted for.

(a)

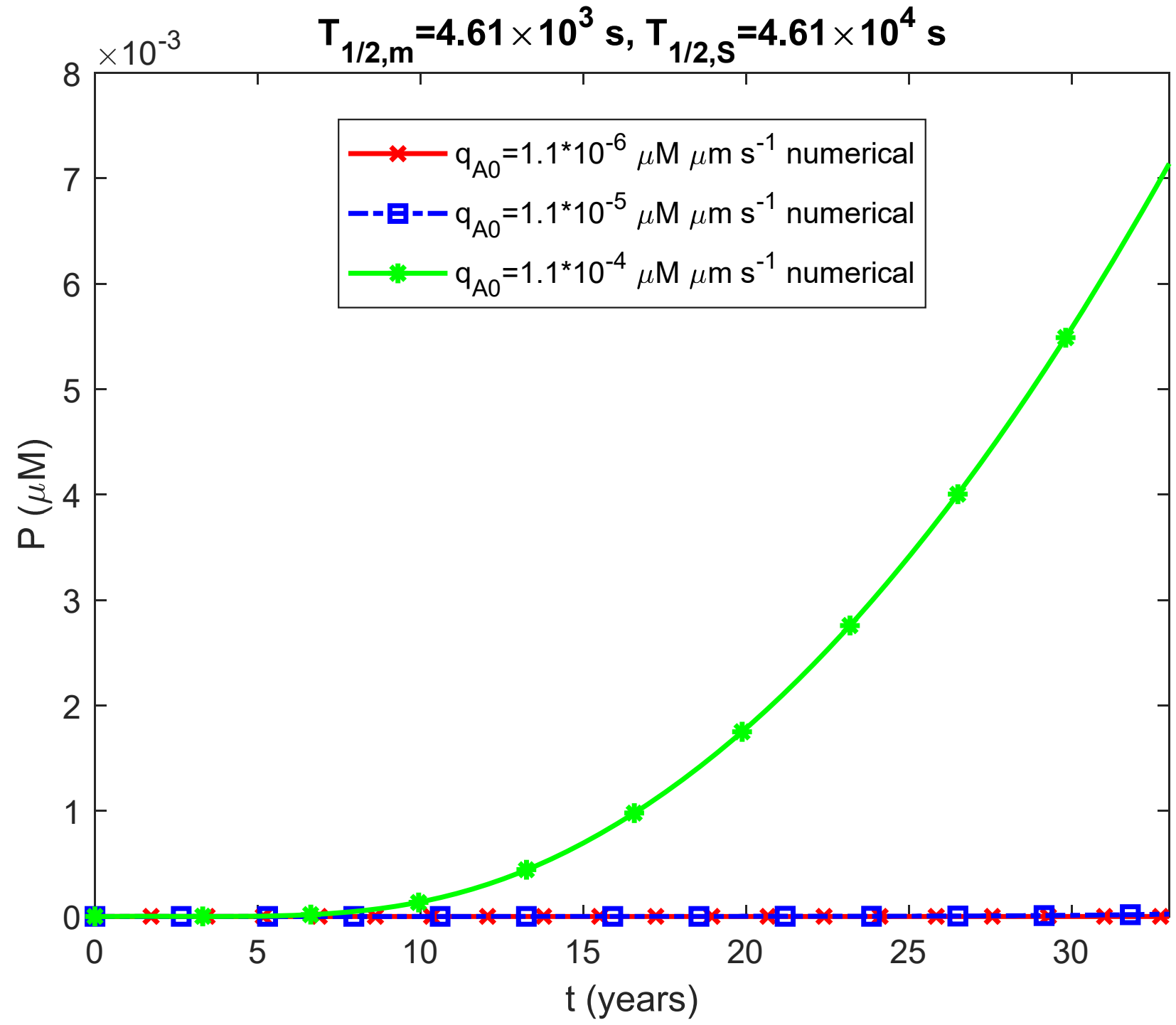

(b)

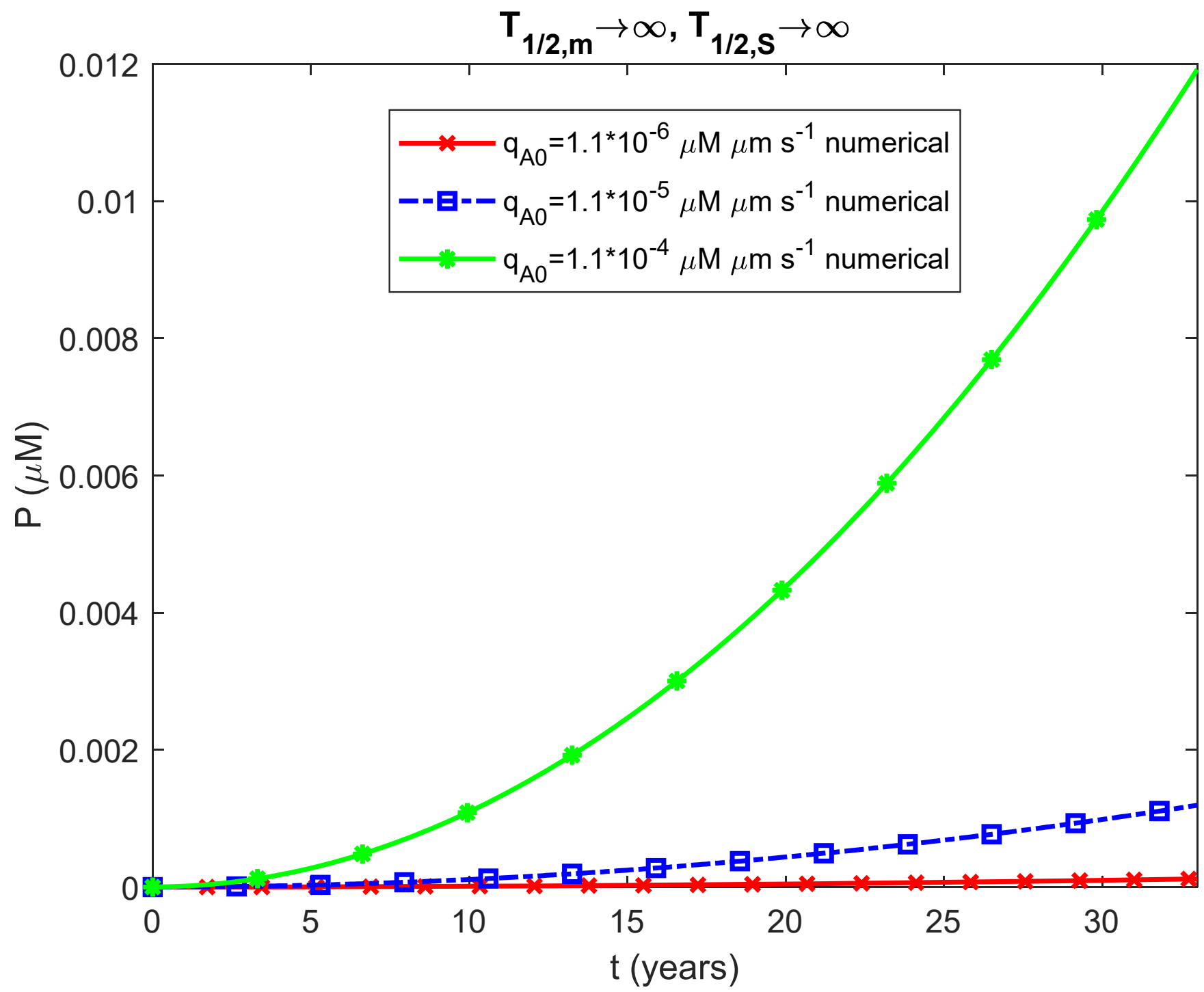

Fig. S12. Molar concentration of Aβ fibrillar species of varying length as a function of time, $P(t)$. The case in which both oligomer dissociation into monomers ($k_{d1} = 3\times10^{-6}$ s^{-1}, $k_{d2} = 1.94\times10^{-5}$ μM^{-1} s^{-1}) and fibril fragmentation ($k_{-} = 10^{-14}$ s^{-1}) are accounted for.

(a)

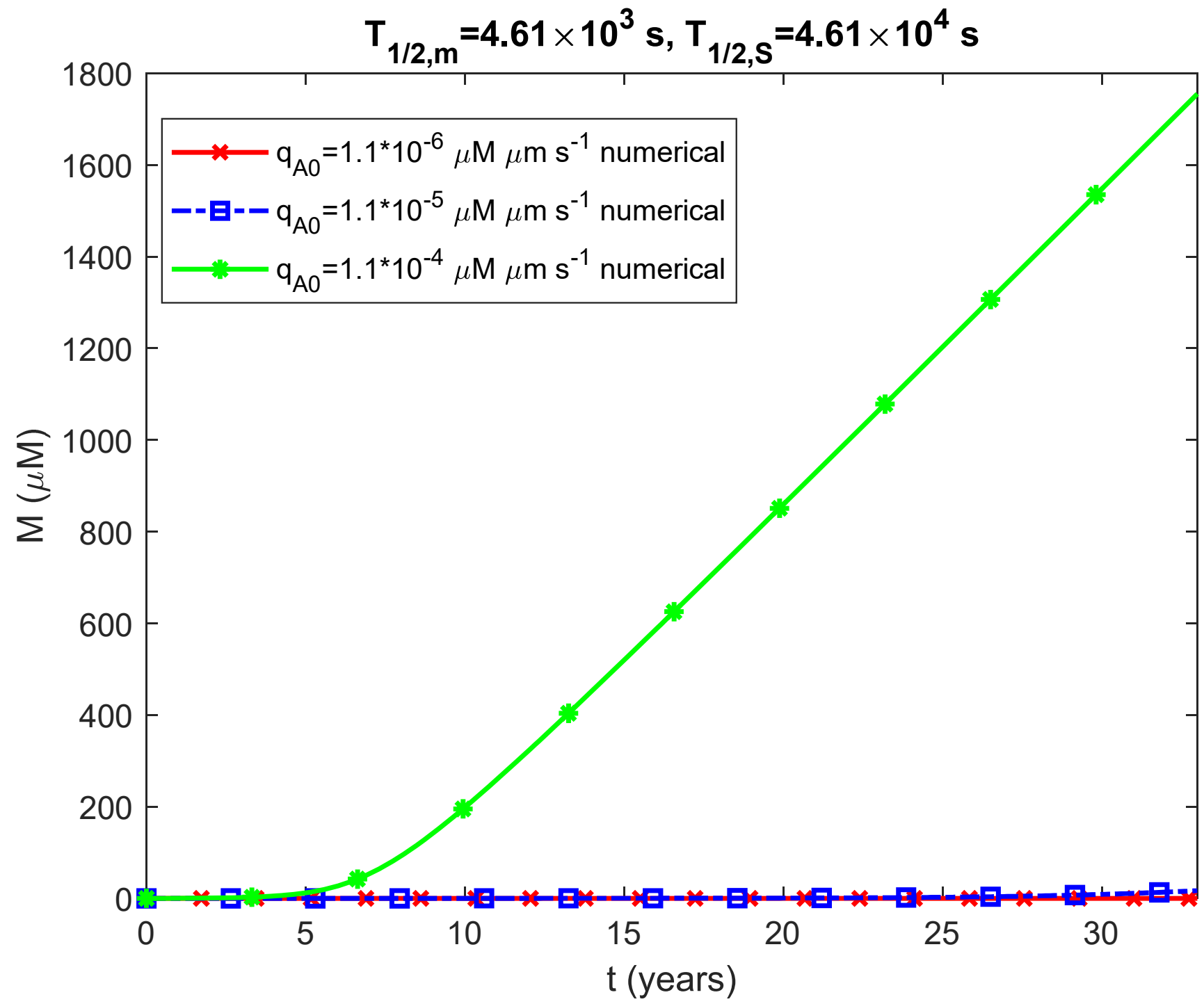

T1/2,m=4.61×10^3 s, T1/2,S=4.61×10^4 s
qA0=1.1*10^-6 μM μm s^-1 numerical
qA0=1.1*10^-5 μM μm s^-1 numerical
qA0=1.1*10^-4 μM μm s^-1 numerical
M (μM)
t (years)

(b)

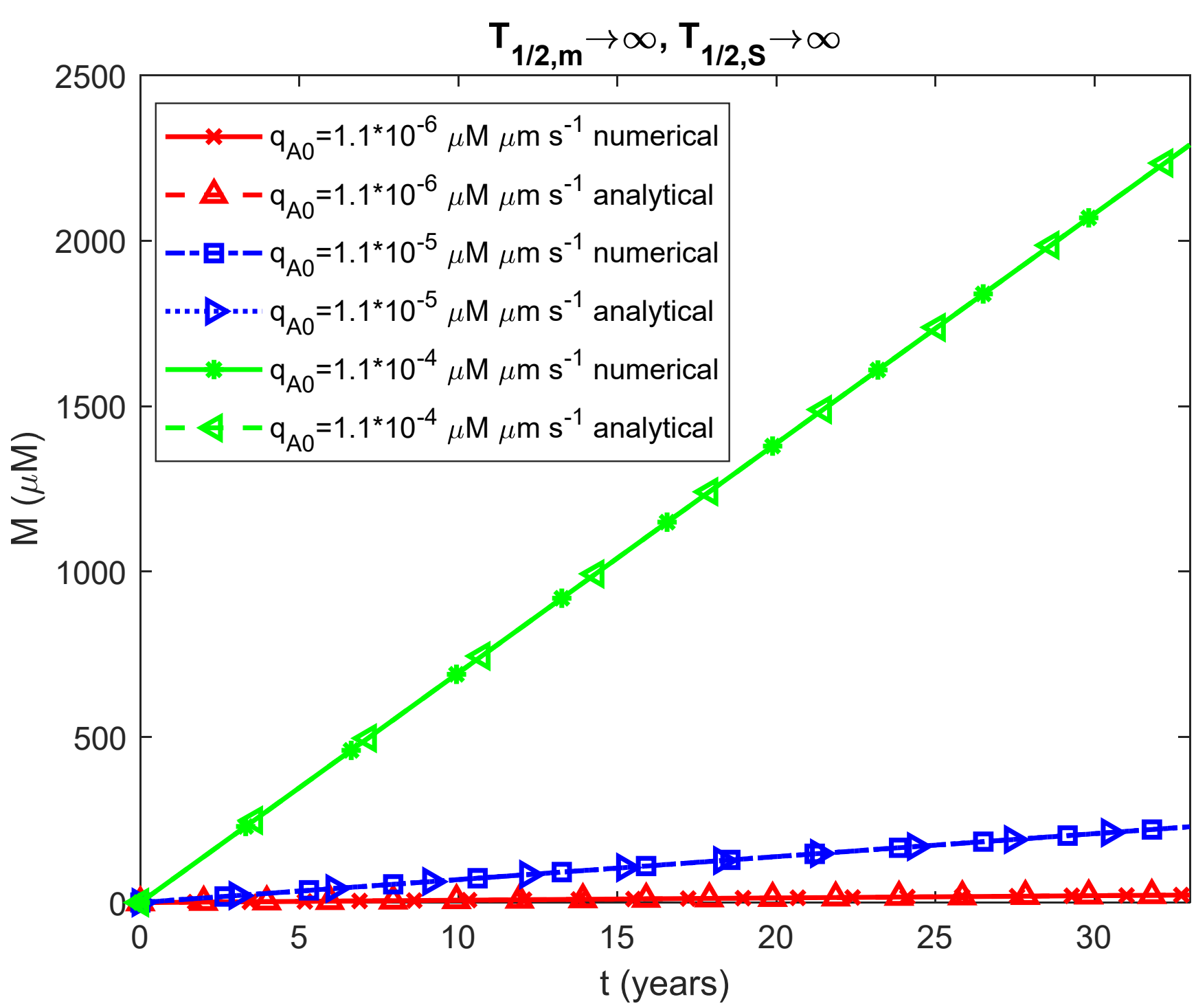

T1/2,m→∞, T1/2,S→∞
qA0=1.1*10^-6 μM μm s^-1 numerical
qA0=1.1*10^-6 μM μm s^-1 analytical
qA0=1.1*10^-5 μM μm s^-1 numerical
qA0=1.1*10^-5 μM μm s^-1 analytical
qA0=1.1*10^-4 μM μm s^-1 numerical
qA0=1.1*10^-4 μM μm s^-1 analytical
M (μM)
t (years)

Fig. S13. Molar concentration of Aβ monomers incorporated into fibrillar species of varying length as a function of time, $M(t)$. The case in which both oligomer dissociation into monomers ($k_{d1}=3\times10^{-6}$ s^{-1}, $k_{d2}=1.94\times10^{-5}$ μM^{-1} s^{-1}) and fibril fragmentation ($k_{-}=10^{-14}$ s^{-1}) are accounted for.

(a)

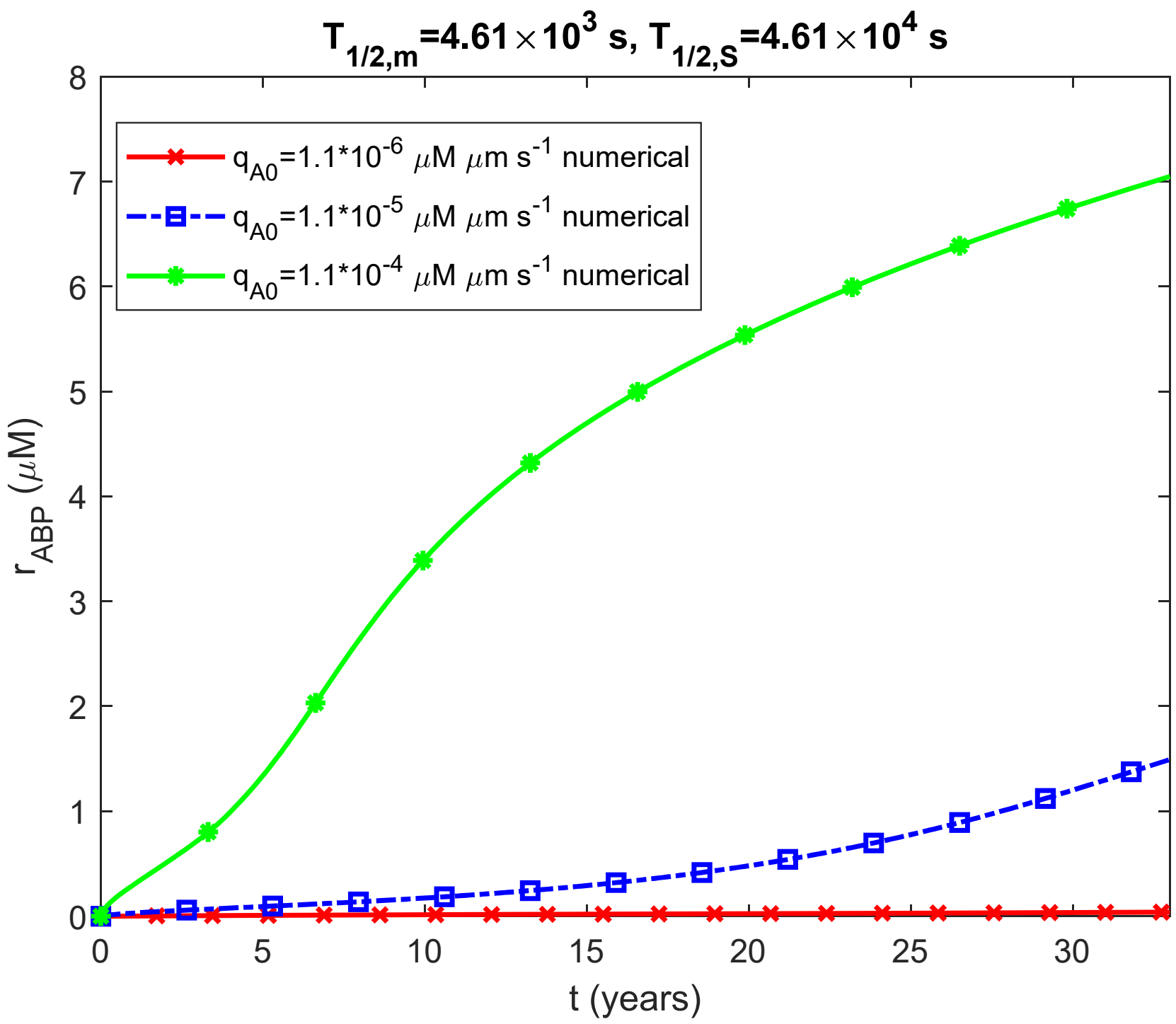

(b)

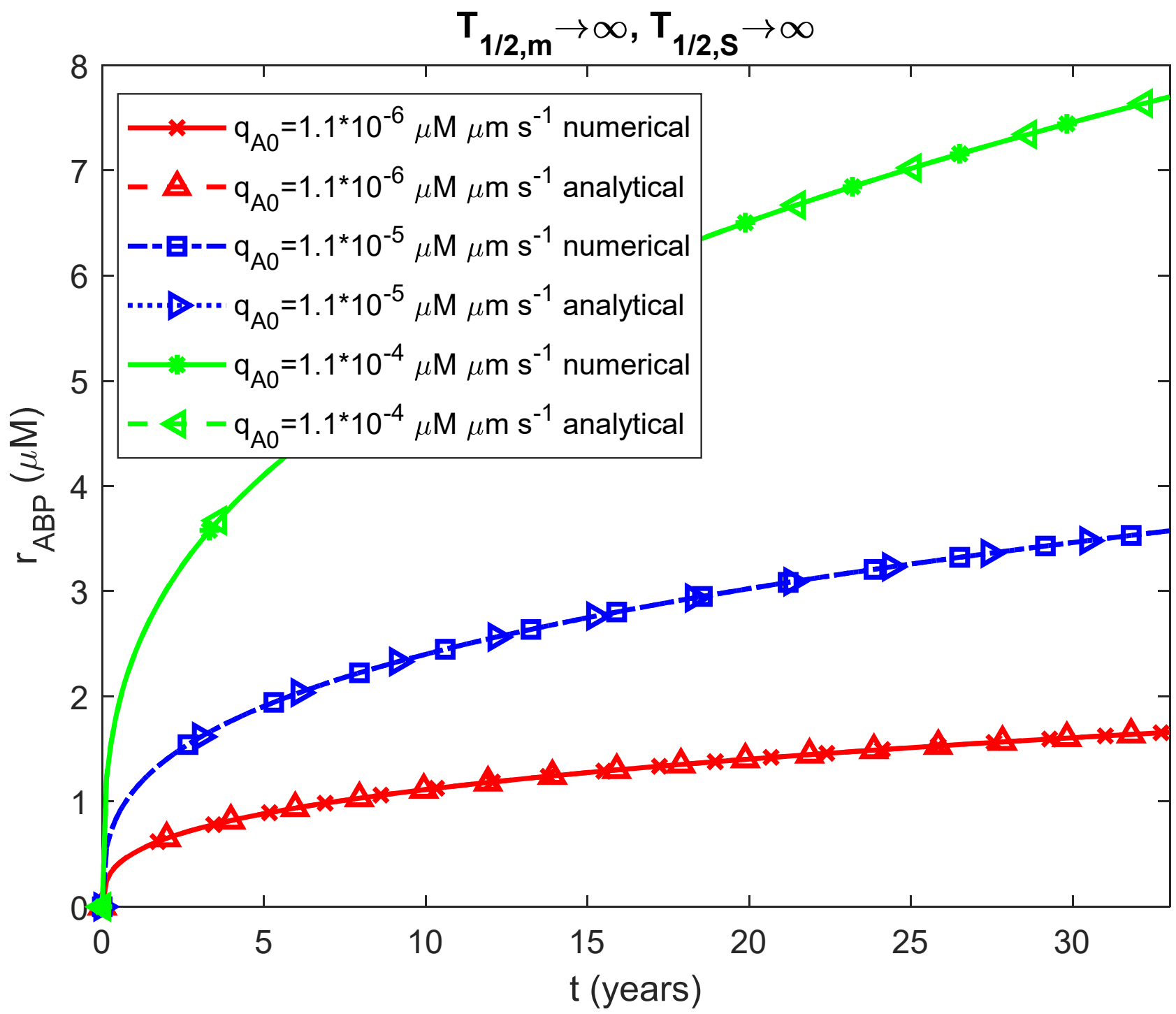

Fig. S14. Radius of the Aβ plaque as a function of time, $r_{ABP}(t)$. The case in which both oligomer dissociation into monomers ($k_{d1} = 3\times10^{-6}$ s^{-1}, $k_{d2} = 1.94\times10^{-5}$ μM^{-1} s^{-1}) and fibril fragmentation ($k_{-} = 10^{-14}$ s^{-1}) are accounted for.

(a)

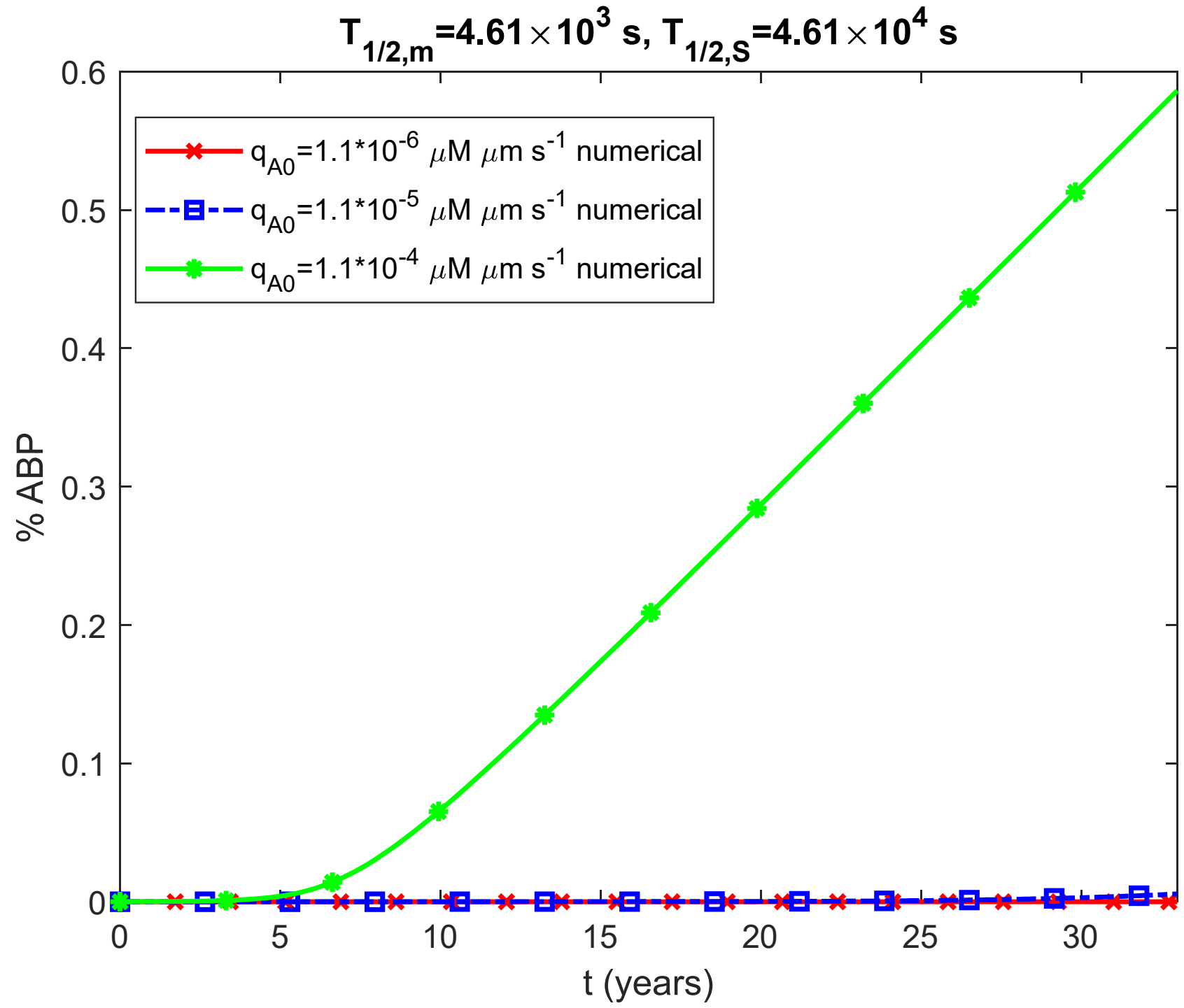
T1/2,m=4.61×10^3 s, T1/2,S=4.61×10^4 s
qA0=1.1*10^-6 μM μm s^-1 numerical
qA0=1.1*10^-5 μM μm s^-1 numerical
qA0=1.1*10^-4 μM μm s^-1 numerical
% ABP
t (years)

(b)

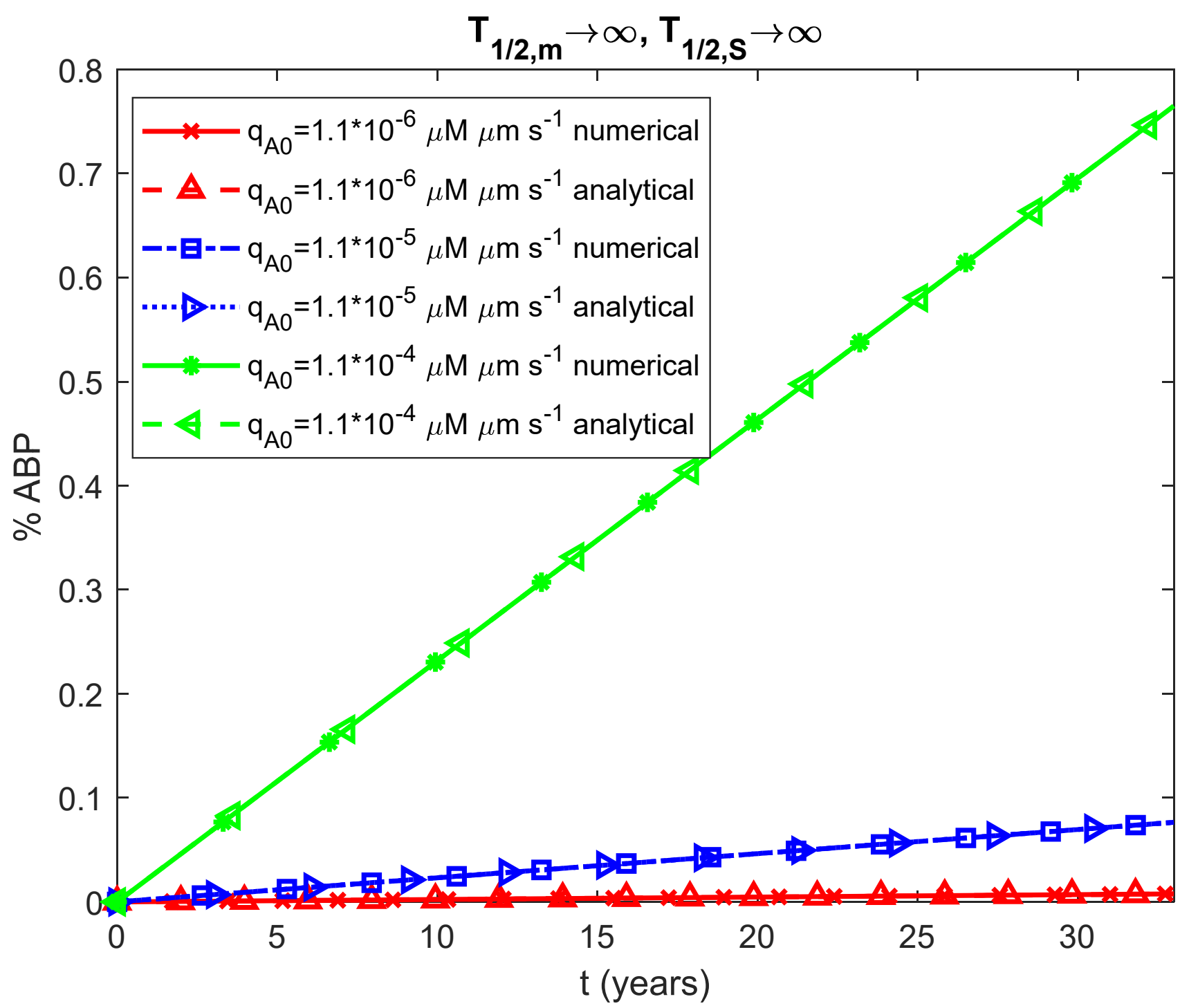
T1/2,m→∞, T1/2,S→∞
qA0=1.1*10^-6 μM μm s^-1 numerical
qA0=1.1*10^-6 μM μm s^-1 analytical
qA0=1.1*10^-5 μM μm s^-1 numerical
qA0=1.1*10^-5 μM μm s^-1 analytical
qA0=1.1*10^-4 μM μm s^-1 numerical
qA0=1.1*10^-4 μM μm s^-1 analytical
% ABP
t (years)

Fig. S15. Percentage of the CV occupied by the Aβ plaque as a function of time. The case in which both oligomer dissociation into monomers ($k_{d1} = 3\times10^{-6}$ s^{-1}, $k_{d2} = 1.94\times10^{-5}$ μM^{-1} s^{-1}) and fibril fragmentation ($k_{-} = 10^{-14}$ s^{-1}) are accounted for.

(a)

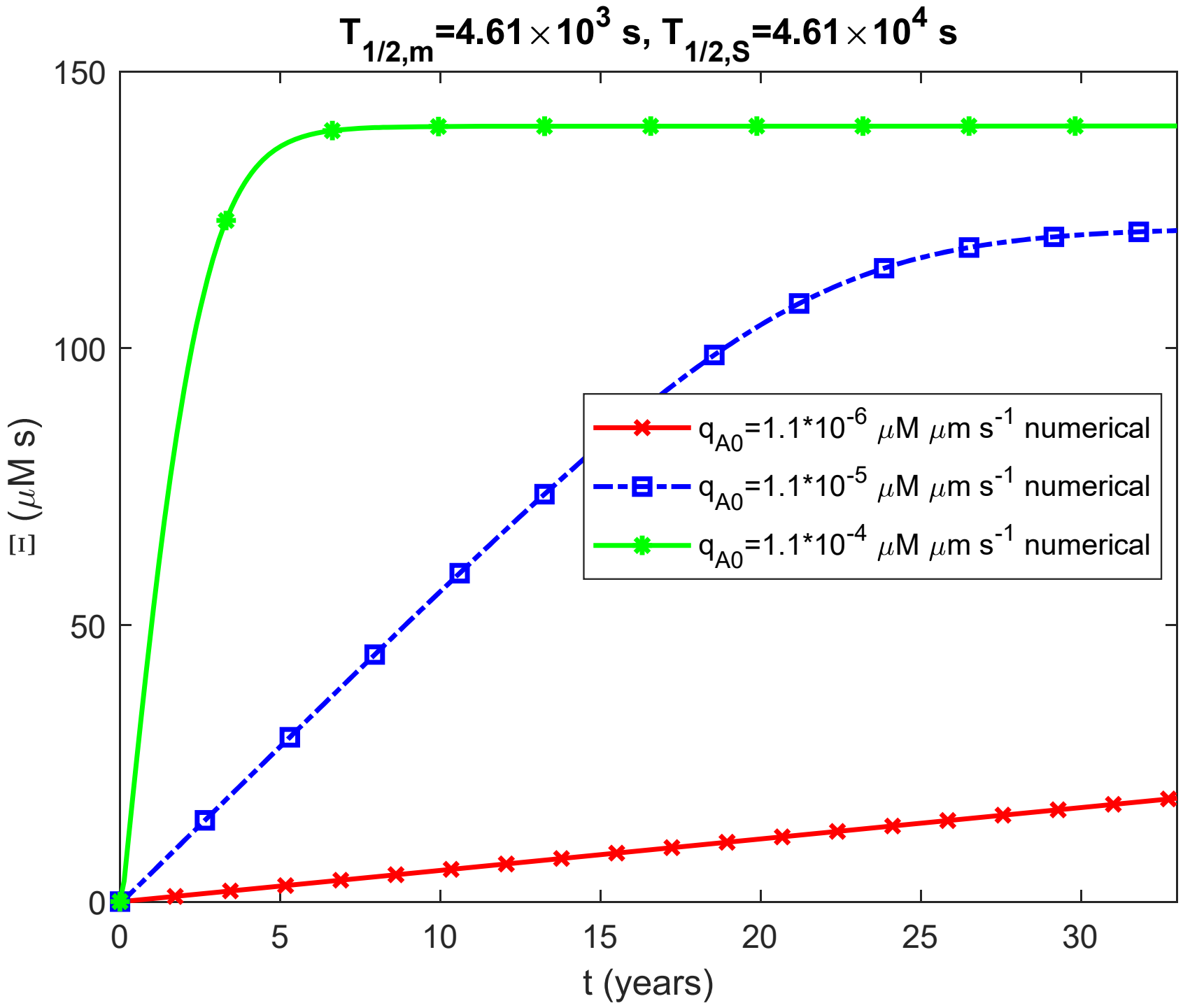

(b)

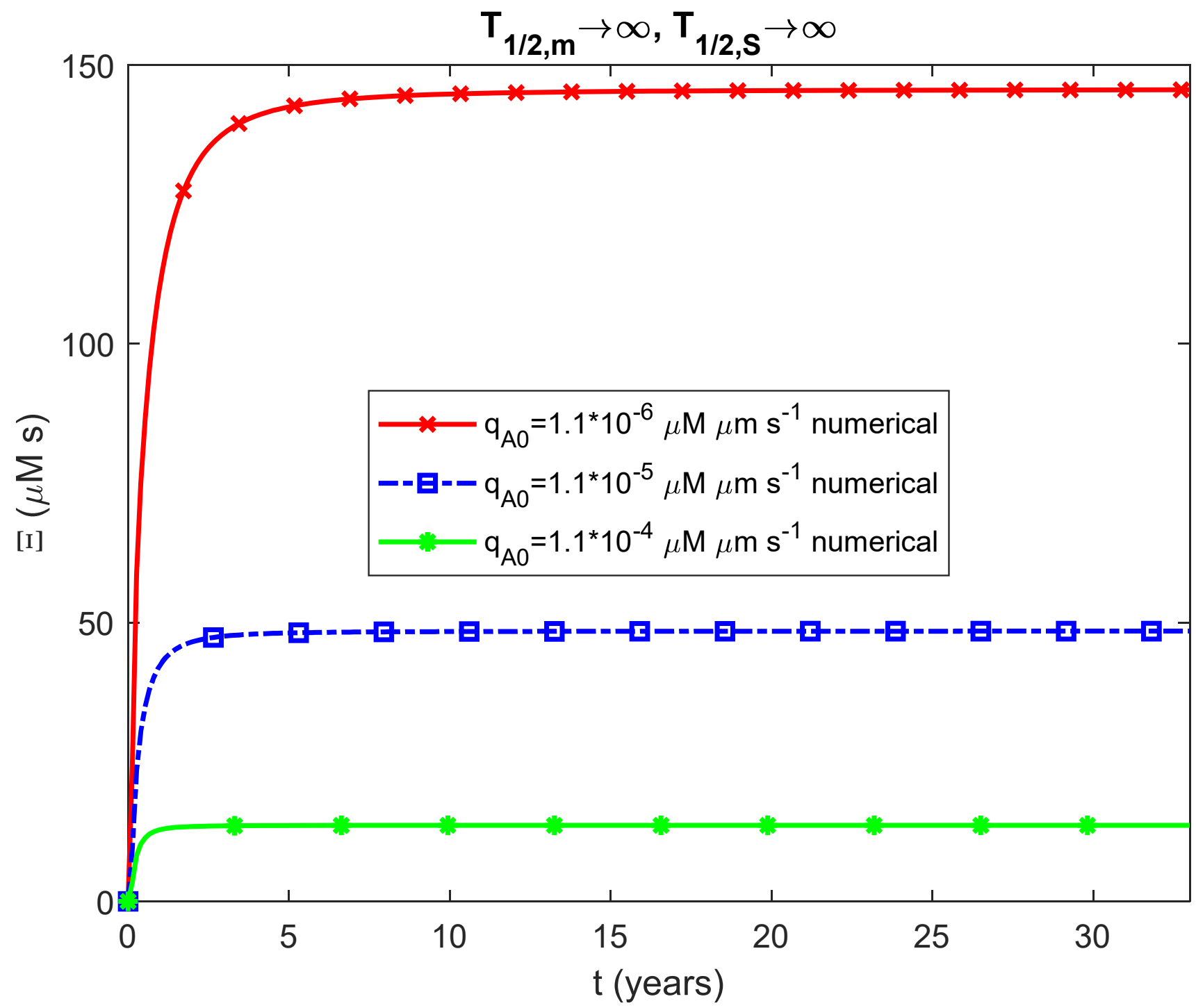

Fig. S16. Accumulated neurotoxicity as a function of time, $\Xi(t)$. The case in which both oligomer dissociation into monomers ($k_{d1}=3\times10^{-6}$ s^{-1}, $k_{d2}=1.94\times10^{-5}$ μM^{-1} s^{-1}) and fibril fragmentation ($k_{-}=10^{-14}$ s^{-1}) are accounted for.

(a)

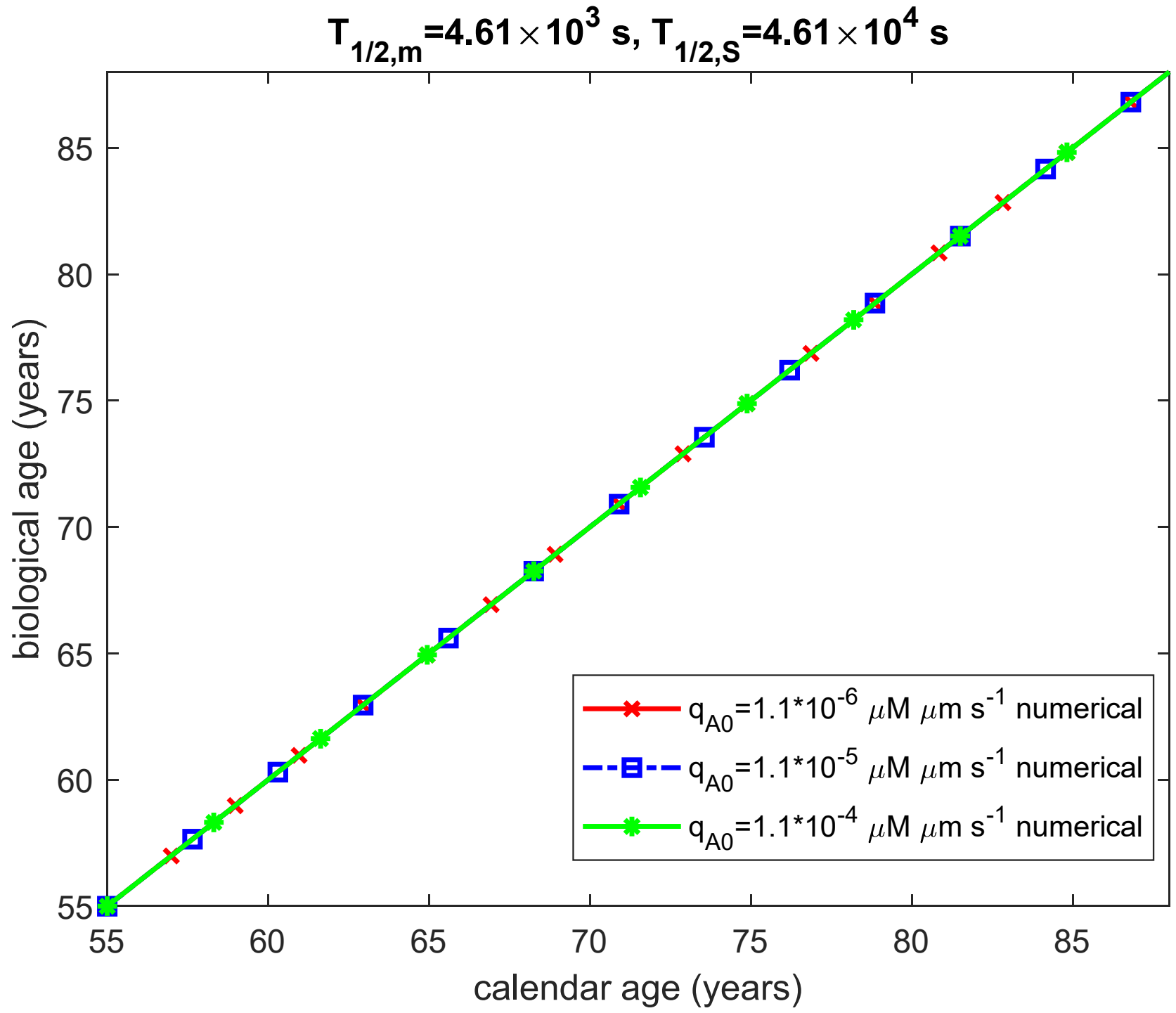
$T_{1/2,m}$=4.61×10^3 s, $T_{1/2,S}$=4.61×10^4 s
biological age (years)
calendar age (years)
55
60
65
70
75
80
85
q_{A0}=1.1*10^-6 μM μm s^-1 numerical
q_{A0}=1.1*10^-5 μM μm s^-1 numerical
q_{A0}=1.1*10^-4 μM μm s^-1 numerical

(b)

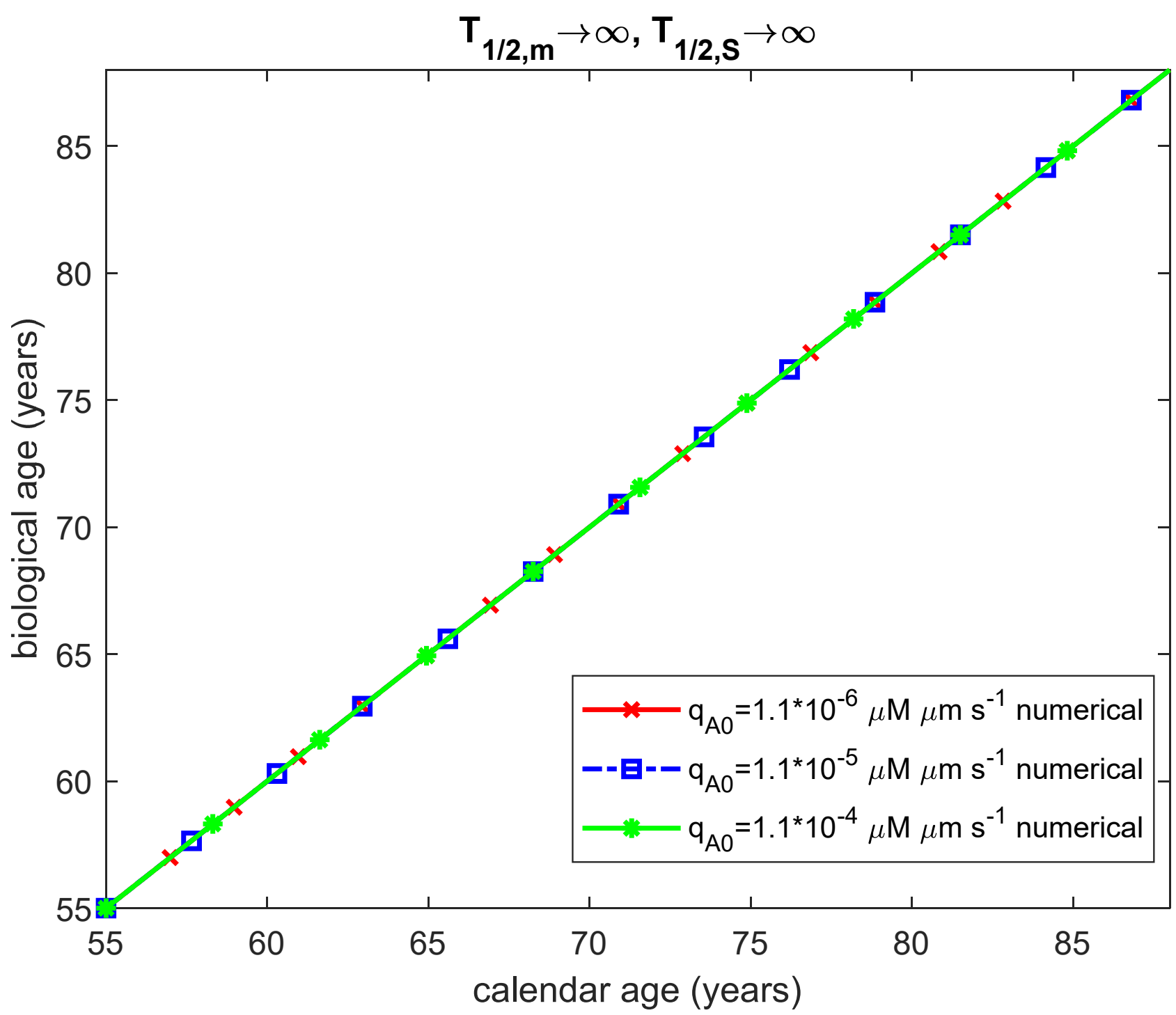
$T_{1/2,m}$→∞, $T_{1/2,S}$→∞
biological age (years)
calendar age (years)
55
60
65
70
75
80
85
q_{A0}=1.1*10^-6 μM μm s^-1 numerical
q_{A0}=1.1*10^-5 μM μm s^-1 numerical
q_{A0}=1.1*10^-4 μM μm s^-1 numerical

Fig. S17. Biological age as a function of calendar age. The case in which both oligomer dissociation into monomers ($k_{d1} = 3\times10^{-6}$ s^{-1}, $k_{d2} = 1.94\times10^{-5}$ μM^{-1} s^{-1}) and fibril fragmentation ($k_{-} = 10^{-14}$ s^{-1}) are accounted for.

(a)

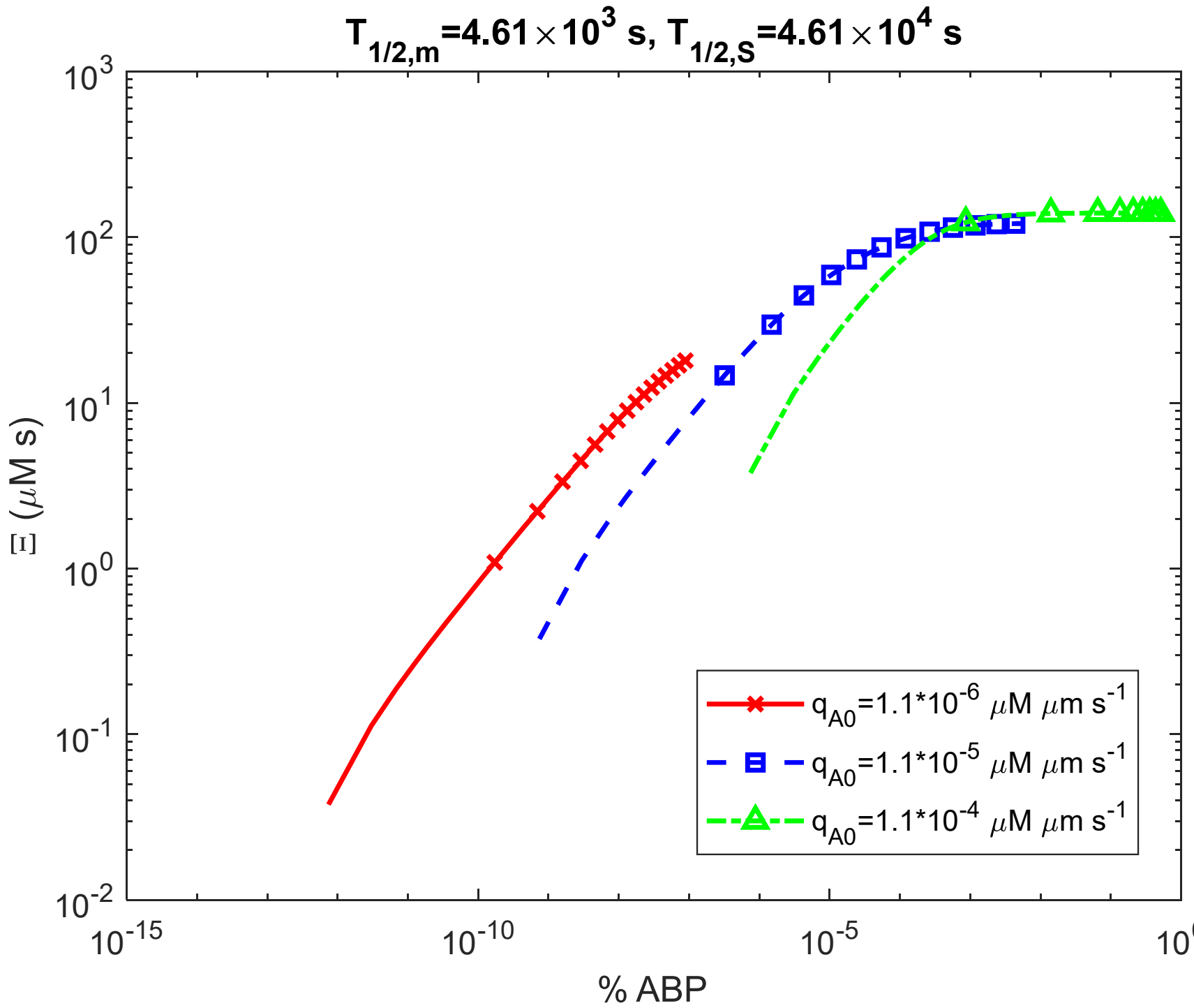

(b)

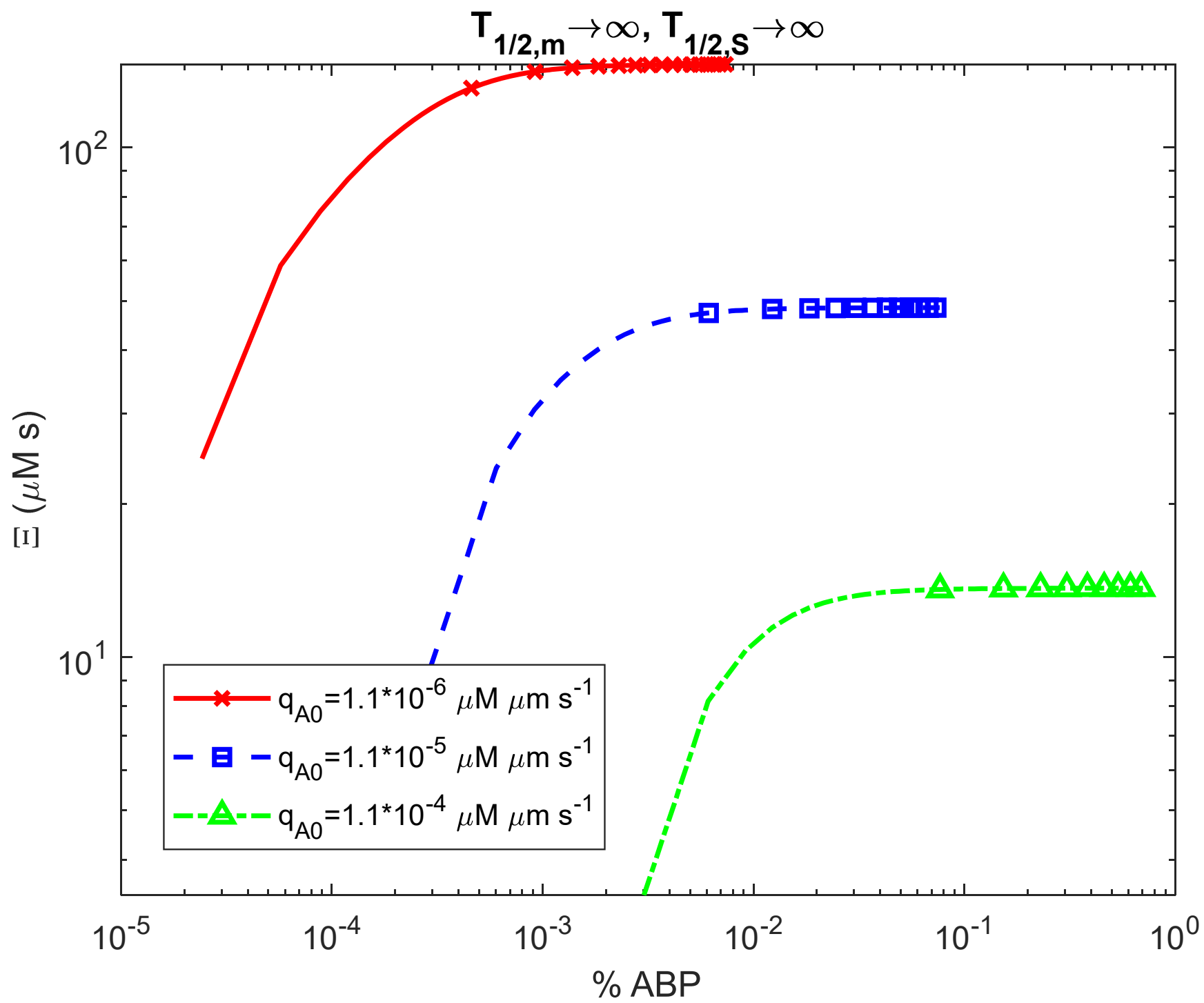

Fig. S18. Accumulated neurotoxicity as a function of the percentage of the CV occupied by the Aβ plaque. The case in which both oligomer dissociation into monomers ($k_{d1} = 3\times10^{-6}$ s^{-1}, $k_{d2} = 1.94\times10^{-5}$ μM^{-1} s^{-1}) and fibril fragmentation ($k_{-} = 10^{-14}$ s^{-1}) are accounted for.
